# (Oxidopyridyl)Porphyrins of Different Lipophilicity: Photophysical Properties, ROS Production and Phototoxicity on Melanoma Cells Under CoCl_2_-Induced Hypoxia

**DOI:** 10.3390/antiox14080992

**Published:** 2025-08-13

**Authors:** Martina Mušković, Martin Lončarić, Ivana Ratkaj, Nela Malatesti

**Affiliations:** 1 Faculty of Biotechnology and Drug Development, University of Rijeka, Radmile Matejčić 2, 51000 Rijeka, Croatia; martina.muskovic@biotech.uniri.hr; 2 Laboratory for Photonics and Quantum Optics, Division of Experimental Physics, Ruđer Bošković Institute, Bijenička Cesta 54, 10000 Zagreb, Croatia; martin.loncaric@irb.hr

**Keywords:** (oxidopyridyl)porphyrin, lipophilicity, reactive oxygen species, singlet oxygen, phototoxicity, hypoxia, melanoma cells

## Abstract

One of the main limitations of photodynamic therapy (PDT) is hypoxia, which is caused by increased tumour proliferation creating a hypoxic tumour microenvironment (TME), as well as oxygen consumption by PDT. Hypoxia-activated prodrugs (HAPs), such as molecules containing aliphatic or aromatic *N*-oxide functionalities, are non-toxic prodrugs that are activated in hypoxic regions, where they are reduced into their cytotoxic form. The (oxido)pyridylporphyrins tested in this work were synthesised as potential HAPs from their AB_3_ pyridylporphyrin precursors, using *m*-chloroperbenzoic acid (*m*-CPBA) as an oxidising reagent. Their ground-state and excited-state spectroscopic properties, singlet oxygen (^1^O_2_) production by the photodegradation of 1,3-diphenylisobenzofurane (DPBF) and theoretical lipophilicity were determined. In vitro analyses included cellular uptake, localisation and (photo)cytotoxicity under normoxia and CoCl_2_-induced hypoxia. The CoCl_2_ hypoxia model was used to reveal their properties, as related to HIF-1 activation and HIF-1α accumulation. (Oxido)pyridylporphyrins showed promising properties, such as the long lifetime of the excited triplet state, a high quantum yield of intersystem crossing, and high production of ROS/^1^O_2_. Lower cellular uptake resulted in an overall lower phototoxicity of these *N*-oxide porphyrins in comparison to their *N*-methylated analogues, and both porphyrin series were less active on CoCl_2_-treated cells. (Oxido)pyridylporphyrins showed higher selectivity for pigmented melanoma cells, and the antioxidant activity of melanin pigment seemed to have a lower impact on their PDT activity compared to their *N*-methylated analogues in both CoCl_2_-induced hypoxia and normoxia. Their potential HAP activity will be evaluated under conditions of reduced oxygen concentration in our future studies.

## 1. Introduction

As early as 1903, Herman von Tappeiner and Albert Jesionek recognised the potential of photodynamic action, which arises from the combination of using a dye with light for the treatment of skin tumours [1]. The term ‘photodynamic’ was introduced here and then clearly defined a few years later by von Tappeiner and Albert Jodlbauer, after they discovered that a third component—molecular oxygen—was also necessary to complete this action [1]. Two possible mechanisms in photodynamic therapy (PDT) are now known; these can lead to the formation of singlet oxygen (^1^O_2_) and other reactive oxygen species (ROS), thereby resulting in oxidative stress, which is detrimental to the cell in which it is generated [2]. For both mechanisms, the population of the triplet excited state of the photosensitiser (^3^PS*) is important; this is formed after the excitation of an organic molecule by the absorption of light and intersystem crossing (ISC) from the singlet excited state (^1^PS*). In the type II process, there is a transfer of energy, moving directly from ^3^PS* to oxygen (^3^O_2_), with the consequent formation of ^1^O_2_, while in the type I reaction, PS reacts with the surrounding biomolecules, which involves hydrogen and electron transfer and the formation of radicals and radical ions. These radicals then react with ^3^O_2_, and various ROS are formed, such as the very reactive hydroxyl radical (^•^OH), superoxide radical (O_2_^•−^), and hydrogen peroxide (H_2_O_2_) [2].

Photodynamic therapy has a great advantage over other phototherapies and chemotherapy because the drug photosensitiser (PS) is non-toxic without a light source and is excited by the local application of light only to the tumour tissue in which the PS selectively accumulates, which, in turn, protects the surrounding, healthy tissue. However, one of the limiting factors of PDT is the presence of oxygen molecules. One of the hallmarks of solid tumours like melanoma is their fast proliferation, causing lower oxygen availability and hypoxic conditions in the tumour microenvironment (TME). Photodynamic therapy further influences oxygen concentration by consuming the already limited amount of oxygen [3]. Hypoxic conditions can be very beneficial for tumour survival because they activate the hypoxia induction factor (HIF) family of proteins, which are responsible for the adaptation of cancer cells to conditions of reduced oxygen concentration. They regulate around 100 genes that are involved in metabolic pathways, angiogenesis, DNA replication and protein synthesis, which results in greater cell proliferation, invasion and migration [4]. Activated HIF-1 plays a major role in these events, and increased levels of HIF-1 correlate with higher tumour metastatic potential, neoangiogenesis, and the development of resistance to cancer therapies [5]. The HIF-1α protein is its subunit; in hypoxia, this becomes stable and is expressed throughout the body, and there are several clinical trials in different phases with drugs that target the HIF-1α pathway for cancer therapy [3]. Hypoxia and increased HIF-1 activity are also associated with cancer aggressiveness, metastasis and resistance in melanoma [6]. For example, pazopanib, which is a HIF-targeting drug, is currently undergoing clinical trials against melanoma [3]. Another approach for cancer therapy that targets hypoxia involves hypoxia-activated prodrugs (HAPs), such as tirapazamine and banoxantrone, which are otherwise non-toxic compounds with aromatic or aliphatic tertiary amine *N*-oxide groups that can be reduced by hypoxia to their cytotoxic form [7]. Banoxantrone, also known as AQ4N, was investigated in combination with PDT, and there are several examples of studies where tirapazamine was used as an HAP with PSs such as Chlorin e6 (Ce6), often with other agents for additional functionalities, within complex mixtures, nanomaterials, liposomes, etc., for combination therapy with PDT [8]. However, such combinations have not yet entered clinical use, and the HAPs themselves have not yet met their high expectations [9].

In addition to treating resistant cancers and metastases, reaching deep-seated tumours is also often mentioned as a great challenge for PDT. Rather, it is increasingly emphasised that striving for an ideal photosensitiser that should fully meet all the requirements from a well-known long list may not be the best approach to address these limitations; moreover, this probably slows down the wider use of PDT in the clinic setting [10]. Among the most important criteria for PS, it is certainly necessary to strive for efficient ^3^PS* formation and the high production of ^1^O_2_, whereas for application in hypoxic conditions, it is desirable to have a PS that can employ a hypoxic environment for the same purpose or even for additional anti-cancer action by other mechanisms. Insisting on the high absorption of red light by a PS may not be as important for application on superficial lesions, but for clinical applications, it might be better to develop a PS for use as a single agent with good selectivity and effective entry into tumour cells, rather than developing complex mixtures of agents that are expected to exert different functions simultaneously [10]. Certainly, preference should be given to those PSs that can also elicit an immune response for a more comprehensive overall effect [11]. Therefore, in the development of PSs, the type of tumour and tumour cells to be treated should be considered from the beginning, and the PS should be developed together with appropriate irradiation conditions and dosimetry.

Melanoma, the deadliest type of skin cancer, especially if it is advanced, is still a huge challenge for all current therapies, and its TME and mechanisms of resistance are still not sufficiently understood [12]. The role of oxidative stress in melanoma is complex and can have quite opposite effects on the progression of the disease, due to the mechanisms that melanoma uses to protect itself and reduce oxidative stress in the TME. Nevertheless, it seems that antioxidants may play a more beneficial role in prevention, while oxidative stress-inducing agents are proving to offer more promising approaches for melanoma therapy [13]. However, given the continuous oxidative stress seen in the TME and the levels of ROS, which are higher in melanoma than in normal cells, in order to achieve apoptosis and the destruction of cancer cells, it is necessary to significantly increase the ROS levels during the additional induction of oxidative stress. Photosensitisers with a high production of ^1^O_2_ oxygen and other ROSs upon irradiation and also with the ability to form ROSs in hypoxia might, therefore, improve the use of PDT against melanoma.

In our previous work, a systematic study of asymmetric (AB_3_) tricationic pyridiniumporphyrins, with the only difference in their structure being different (lipophilic) alkyl chain lengths, our research confirmed the importance of the efficient cellular uptake of PSs for achieving the highest levels of phototoxicity [14]. The lowest *IC*_50_ values on melanoma cells were achieved with amphiphilic porphyrins that showed the highest cellular uptake, i.e., those with the longest alkyl chains (**TMPyP3-C_17_H_35_**, the free base and its Zn(II) complex, after irradiation at 643 and 606 nm, respectively), while those with shorter chains (**TMPyP3-C_13_H_27_** and **Zn(II)-TMPyP3-C_13_H_27_**) showed the highest selectivity for melanoma cells in comparison to fibroblasts [14]. Having both amphiphilic properties and an asymmetric porphyrin structure with a long lipophilic chain, these qualities have been shown to be responsible for efficient cellular entry, indicating a high potential for the passive selectivity of our PSs in PDT and possibly also for active targeting to some extent, due to the specific lipid metabolism of cancer cells [15]. Therefore, we decided to explore the possibility of using *N*-oxide analogues of those PSs with the most effective cellular uptake, for use in PDT on melanoma cells in hypoxic conditions. We chose *N*-oxide functionality because of its easy introduction through pyridyl nitrogen oxidation, and because of its numerous advantages in medicinal chemistry, such as increasing drug polarity and playing a role in HAP. Furthermore, pyridyl *N*-oxides are weak bases, but they are less polar and more stable compared to aliphatic tertiary amine oxides [16]. Amine *N*-oxides are usually very hygroscopic, but this is less marked with an increase in lipophilicity in the molecule [17]. They can be used for targeting hypoxia and cancer tissue, where they can undergo one- or two-electron enzymatic reduction, which may result in the formation of cytotoxic species, prodrug radical anions, hydroxyl radical and deoxygenated (parent) amines [16]. In the case of tirapazamine, bioreduction leads to apoptosis and HIF-1α downregulation in cancer cells. However, when working in vivo with this HAP, certain drawbacks have been revealed, such as its low bioavailability and adverse effects; therefore, tissue-penetrating analogues are being sought [18].

Consequently, the present study endeavours to prepare several porphyrins with *N*-oxide moieties of different lipophilicity and to investigate their activity as PSs in melanoma cells in comparison to our previously studied *N*-methylated analogues. We decided to use cobalt(II) chloride for hypoxia induction in melanoma cells to investigate, firstly, the impact of the increased HIF-1α on the phototoxicity of PSs. Although CoCl_2_ only mimics lowered oxygen concentrations, its influence is sufficient for the effective blocking of HIF-1α degradation and activating hypoxic changes that correlate to real conditions [19]. The results of our study have shown that (oxido)pyridylporphyrins have favourable properties for use as PSs in PDT, with a long lifetime for the excited triplet state (*τ*_T_) and a high quantum yield of intersystem crossing (*Φ*_ISC_). The lower cellular uptake of (oxido)pyridlporphyrins compared to their *N*-methylated analogues resulted in lower phototoxicity. However, in contrast to *N*-methylated analogues, melanin and its antioxidant activity had minimal or no effect on the photocytotoxicity of (oxido)pyridyporphyrins.

Moreover, the (oxido)pyridylporphyrins showed higher selectivity (SI value) for the pigmented melanoma cell line compared to non-pigmented, suggesting that the *N*-oxide moiety in the pyridylporphyrins may play some role in combating hypoxia and the antioxidant activity of pigment melanin in melanoma cell lines in vitro. Our future work will investigate their HAP potential in other hypoxia models.

## 2. Materials and Methods

### 2.1. Synthesis

#### 2.1.1. General

All reagents were purchased from Sigma Aldrich (St. Louis, MO, USA) or Alfa Aesar (Ward Hill, MA, USA), while solvents were purchased from BDH Prolabo (Bengaluru, India) and GramMol (Zagreb, Croatia). Dry dichloromethane (DCM) was prepared by keeping the solvent over an activated molecular sieve 4 Å (Sigma Aldrich) in a N_2_ atmosphere for at least 24 h before use. Silica gel 40–63 µm (230–400 mesh) and thin-layer chromatography (TLC) plates (0.2 mm) on aluminium foil with a UV_254_ fluorescence indicator were purchased from Macherey-Nagel (Düren, Germany). ^1^H and ^13^C NMR spectra were recorded on a Bruker Advance DPX 600 spectrometer in the Laboratory for NMR Spectroscopy, which is part of the Centre of Excellence in Chemistry at the University of Zagreb. Chloroform-*d*, methanol-*d*_4_, or a mixture of both were used as solvents, and spectra were recorded at 600 MHz for ^1^H NMR and 150 MHz for ^13^C NMR. The spectra results are shown with chemical shifts (*δ*) in parts per million (ppm), relative to tetramethylsilane (TMS, 0 ppm), and with coupling constants (*J*) expressed in Hertz (Hz). Details of ^1^H (for all) and ^13^C NMR spectra (for new) (oxidopyridyl)porphyrins prepared in this work are given in the Supplementary Materials (Figures S1–S7).

Mass spectra of the porphyrins were recorded using high-resolution mass spectrometry (HRMS) on a 6546 LC/Q-TOF (Agilent, Santa Clara, CA, USA), equipped with a high-performance liquid chromatography 1290 Infinity II HPLC (Agilent, Santa Clara, CA, USA) in the Laboratory for Bioanalytics at the Ruđer Bošković Institute (IRB). Samples were dissolved in methanol, and 0.1% trifluoroacetic acid was added. Spectra were recorded in ESI + ionisation mode, and the data were collected at every second in the range of 100–1100 Da.

#### 2.1.2. Preparation of *N*-Methylated Pyridiniumporphyrins (TMPyP3 with an Alkyl Chain) and Their Unmethylated/Unoxidised Precursors (TPyP3 with an Alkyl Chain)

**Figure 1:**
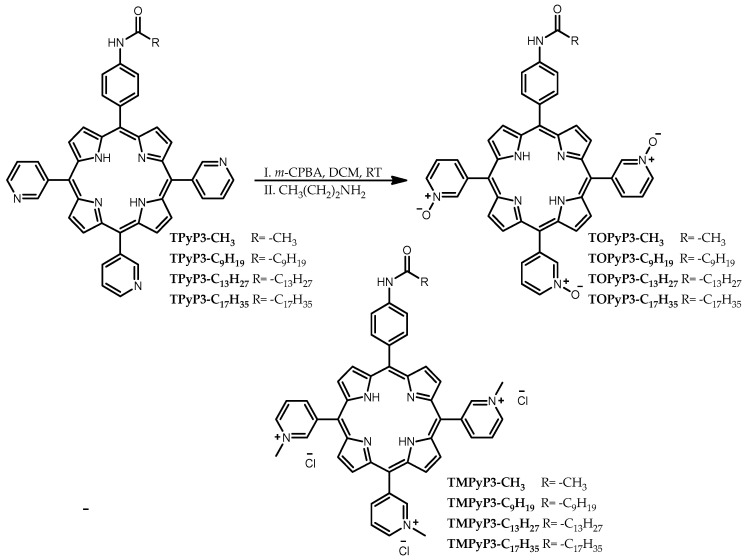
Synthesis of (oxidopyridyl)porphyrins by the pyridyl-*N*-oxidation of (3-pyridyl)porphyrins, and the structure of *N*-methylated pyridiniumporphyrins used in this work.

The porphyrins **TPyP3-CH_3_**, **TPyP3-C_9_H_19_**, **TPyP3-C_13_H_27_**, **TPyP3-C_17_H_35_**, **TMPyP3-CH_3_**, **TMPyP3-C_9_H_19_**, **TMPyP3-C_13_H_27_** and **TMPyP3-C_17_H_35_** (vide infra, Figure 1) were prepared, following the procedures described in our publications [14,20,21].

#### 2.1.3. General Procedure for the Synthesis of (Oxidopyridyl)Porphyrins

Unoxidised precursor porphyrin (**TPyP3** with an alkyl chain of 1, 9, 13 or 17 C atoms) (~50 mg) was dissolved in dry dichloromethane (DCM) (15–20 mL), then *meta*-chloroperoxybenzoic acid (*m*-CPBA) (18–20 equiv.) was added gradually over 30 min under constant stirring, and then stirred for further 15–30 min at room temperature. Upon completion of the reaction, monitored by thin-layer chromatography (DCM: MeOH, 9:1), propylamine (PrA) was added (1–2 mL) and the reaction was stirred for another 30 min at room temperature. The solvent was removed in vacuo, and the reaction mixture was dissolved in DCM. The product was purified twice with column chromatography using DCM and methanol (MeOH) in different ratios. Porphyrins were isolated as a purple or violet solid.

5-(4-Acetamidophenyl)-10,15,20-tris(1-oxido-3-pyridyl)porphyrin (**TOPyP3-CH_3_**).

The general procedure for the synthesis of (oxidopyridyl)porphyrins was followed. The mobile phase (DCM:MeOH) for chromatography for the first column was 10:1 and for the second, 5:1. Yield, 61%; ^1^H NMR (CDCl_3_, 600 MHz): *δ*/ppm −2.94 (s, 2H, pyrrole-N*H*), 2.42 (s, 3H, -C*H*_3_), 7.70–7.79 (m, 3H, Py-5-*H*), 7.80–8.07 (m, 2H, Ar-3,5-*H*), 8.07–8.21 (m, 5H, overlapping Py-6-*H*, Ar-2,6-*H*), 8.69–8.77 (m, 3H, Py-4-*H*), 8.84–9.05 (m, 8H, *β*-*H*), 9.05–9.14 (m, 3H, Py-2-*H*); ^13^C NMR (CDCl_3_, 150 MHz): *δ*/ppm 24.9, 112.8, 113.5, 118.2, 122.3, 124.2, 131.4, 135.2, 136.8, 138.4, 139.0, 140.9, 143.0, 168.7; HRMS: theor. calcd. for C_43_H_30_N_8_O_4_ [M + H]^+^ 723,2463; found 723,2457.

5-(4-Decanamidophenyl)-10,15,20-tris(1-oxido-3-pyridyl)porphyrin (**TOPyP3-C_9_H_19_**).

After following the general procedure, and before the column chromatography, the crude product was dissolved in DCM (20 mL) and the organic layer was washed with H_2_O (3 × 20 mL). After drying over sodium sulphate (Na_2_SO_4_), the salt was removed by filtration, and the solvent was evaporated under pressure. The product was purified twice with column chromatography, with DCM/MeOH 10:1 as a mobile phase. Yield, 73%; ^1^H NMR (CD_3_OD, 600 MHz): *δ*/ppm 0.93 (t, 3H, *J* = 6.9 Hz, -C^10^*H*_3_), 1.24–1.51 (m, 12H, C^4^*H*_2_(C*H*_2_)_4_C^9^*H*_2_), 1.78–1.85 (m, 2H, C^3^*H*_2_), 2.53 (t, 2H, *J* = 7.5 Hz, C^2^*H*_2_), 7.83–8.02 (m, 7H, overlapping Py-5-*H*, Ar-2,6-*H*, Ar-3,5-*H*), 8.25–8.38 (m, 3H, Py-6-*H*), 8.69–9.23 (m, 14H, overlapping Py-4-*H*, *β*-*H*, Py-2-*H*); ^13^C NMR (CD_3_OD, 150 MHz): *δ*/ppm 13.1, 22.3, 25.6, 29.0, 29.1, 29.2, 29.3, 31.7, 36.8, 112.6, 113.2, 118.1, 122.1, 124.9, 134.2, 134.6, 136.2, 138.7, 138.8, 141.1, 142.5, 173.6; HRMS: theor. calcd. for C_51_H_46_N_8_O_4_ [M + H]^+^ 835.3720; found 835.3720.

5-(4-Tetradecanamidophenyl)-10,15,20-tris(1-oxido-3-pyridyl)porphyrin (**TOPyP3-C_13_H_27_**).

The general procedure was followed and, prior to the column chromatography, the crude product was dissolved in H_2_O:trifluoroacetic acid (TFA) (20:1), followed by neutralisation to pH = 8 and precipitation by adding 1M NaOH. The product was extracted with DCM (2 × 20 mL), and the organic layer was further washed with H_2_O (2 × 20 mL). The organic layer was first dried over Na_2_SO_4_, and then the solvent was removed in vacuo. Purification of the product was performed twice with column chromatography: first with 15:1, and then second with 10:1 DCM: MeOH. Yield, 79%; ^1^H NMR (CDCl_3_, 600 MHz): *δ*/ppm −2.94 (s, 2H, pyrrole N*H*), 0.90 (t, 3H, *J* = 7.0 Hz, -C^14^*H*_3_), 1.22–1.56 (m, 20H, C^4^*H*_2_(C*H*_2_)_8_C^13^*H*_2_), 1.87–1.95 (m, 2H, C^3^*H*_2_), 2.60 (t, 2H, *J* = 7.6 Hz, C^2^*H*_2_), 7.69–7.77 (m, 3H, Py-5-*H*), 7.95–8.25 (m, 7H, overlapping Py-6-*H*, Ar-2,6-*H*, Ar-3,5-*H*), 8.72 (d, 3H, *J* = 6.3 Hz, Py-4-*H*), 8.85–9.05 (m, 8H, *β*-*H*), 9.06–9.13 (m, 3H, Py-2-*H*); ^13^C NMR (CDCl_3_, 150 MHz): *δ*/ppm 14.2, 22.7, 25.8, 29.3, 29.5, 29.6, 29.7, 29.8, 31.9, 38.1, 112.8, 113.5, 118.2, 124.3, 131.3, 135.2, 136.6, 138.5, 139.0, 141.0, 143.0, 172.0; HRMS: theor. calcd. for C_55_H_54_N_8_O_4_ [M + H]^+^ 891.4346; found 891.4345.

5-(4-Octadecanamidophenyl)-10,15,20-tris(1-oxido-3-pyridyl)porphyrin (**TOPyP3-C_17_H_35_**).

The crude product was dissolved in H_2_O:TFA (20:1), then 1M NaOH was added to the solution until pH = 8. The precipitate was extracted with DCM (2 × 20 mL), and the organic layer was washed with H_2_O (2 × 20 mL). The organic layer was dried over Na_2_SO_4_, and then the solvent was removed in vacuo. The product was further purified twice by column chromatography, with DCM/MeOH (15:1) used as a mobile phase. Yield, 39%; ^1^H NMR agrees with the previously published data [22]: ^1^H NMR (CDCl_3_ + CD_3_OD, 600 MHz): *δ*/ppm 0.87 (t, 3H, *J* = 6.9 Hz, C^18^*H*_3_), 1.20–1.54 (m, 28H, C^4^*H*_2_(C*H*_2_)_12_C^17^*H*_2_), 1.83–1.91 (m, 2H, C^3^*H*_2_), 2.56 (t, 2H, *J* = 7.6 Hz, C^2^*H*_2_), 7.79–7.87 (m, 3H, Py-5-*H*), 7.89–8.11 (m, 2H, Ar-3,5-*H*), 8.12–8.18 (m, 2H, Ar-2,6-*H*), 8.18–8.28 (m, 3H, Py-6-*H*), 8.71–8.76 (m, 3H, Py-4-*H*), 8.76–9.17 (m, 11H, overlapping *β*-*H* and Py-2-*H*).

### 2.2. Spectroscopy Studies

The absorption spectra of (oxidopyridyl)porphyrins were recorded in HPLC grade MeOH on a Cary UV 60 (Agilent Technologies, Santa Clara, CA, USA), in a wavelength range of 300–700 nm. The fluorescence spectra in HPLC grade MeOH were recorded on a Cary Eclipse (Agilent Technologies) in a wavelength range of 500–800 nm after excitation at the Soret band wavelength (λ~420 nm). The slits of the spectrometer were set to a bandpass of 5 nm for the excitation and 5 nm for the emission. To calculate the fluorescence quantum yields, the measurements were conducted on an FS5 Edinburgh Instruments spectrometer. Prior to taking the measurements, the solutions were purged with high-purity N_2_ for at least 15 min. The absorbance of the solutions was set to *A* < 0.1 at 405 nm and 550 nm, which settings were used for the excitation process. Emission was recorded from 550 to 800 nm, with slits set to the bandpass corresponding to 2 nm for the excitation, and 3 nm for the emission. Fluorescence quantum yield (*Φ*_FL_) was calculated in comparison to the tetraphenylporphyrin (**TPP**; *Φ*_FL_ = 0.11 [23]) as a standard, according to Equation (S1) and following an equation previously described elsewhere [24].

Time-correlated single-photon counting (TC-SPC) was used to obtain information about fluorescence decays. All measurements were performed on an FS5 Edinburgh Instruments device with a pulsed laser at 405 nm wavelength, which was used for the excitation process (pulse duration 60 ps). Fluorescence signals were monitored at 660 nm over 1023 channels, with a time increment of ≈49 ps/channel. Porphyrins were dissolved in MeOH (HPLC grade), with the absorbance adjusted to 0.1 a.u. at the excitation wavelength, and the solution was degassed with N_2_ for 15 min before the measurements were taken. All the decays were collected until they reached 3000 counts in the peak channel. A scattering solution, a silica gel suspension in H_2_O, was used to obtain the instrument response function (IRF). The histograms were analysed using a nonlinear least-squares deconvolution method via the software implemented with the instrument [24].

Laser flash photolysis (LFP) was performed using an LP980 transient absorption spectrometer (Edinburgh Instruments) equipped with a continuous beam (or pulse mode) Xenon lamp passing through the sample. A nanosecond Nd:YAG laser (Quantel, Q-smart 450, 5 ns pulse duration, 10 Hz, excitation wavelength 355 nm) was then used to excite the sample perpendicular to the lamp beam. The laser beam energy was adjusted to below 5 mJ. The ground state absorbance of the porphyrins in MeOH was adjusted to 0.20–0.25 at 355 nm (the laser excitation wavelength) and the solutions were purged with high-purity N_2_ for at least 15 min prior to taking the measurements. The samples were prepared for *k*_q_ determination in an air atmosphere that was N_2_-purged for 15 min and O_2_-purged for 15 min before the measurements were taken. The ground state absorption spectra of the samples were also recorded after purging the sample with N_2_ or O_2_, and after transient absorption (TAS) measurements were taken to track the differences in every step of the measurement process to ensure that no photoproducts were formed. All measurements were performed at 25 °C in 1 cm quartz cuvettes. Laser flash photolysis was used to measure the kinetics of triplet state depopulation (*τ*_T_) and triplet-triplet absorption spectra, and to calculate the intersystem crossing quantum yield (*Φ*_ISC_) and quenching rate constants of triplet excited states by molecular oxygen (*k*_q_). To calculate *Φ*_ISC_, the first step was the calculation of the molar absorption coefficient of the triplet state using the singlet depletion method [25], completing the first step according to Equation (S2). The *Φ*_ISC_ values were estimated by using a comparative method with **TPP** (*Φ*_ISC_ = 0.87 in toluene [23]) as the standard [26]. The *Φ*_ISC_ was calculated according to Equation (S3). Triplet excited state lifetimes were determined by fitting the triplet decays to the equation for the mixed first- and second-order decay, as previously described [24], and the quenching rate constant of a triplet excited state by molecular oxygen was calculated using the Stern–Volmer equation (Equation (S4)).

### 2.3. Reactive Oxygen Species (ROS) and Singlet Oxygen (^*1*^O_*2*_) Measurements

Photodegradation of the fluorescent dye 1,3-diisophenylbenzofurane (DPBF) was used to evaluate and compare the production of reactive oxygen species (ROS) and singlet oxygen (^1^O_2_) by (oxidopyridyl)porphyrins and *N*-methylated pyridiniumporphyrins after light irradiation. Stock solutions of DPBF and porphyrins were prepared in dimethylsulfoxide (DMSO) and diluted in MeOH. The same volumes (1 mL) of the porphyrin (10 µM) and DPBF (8 µM) solutions were mixed together in a cuvette until reaching a final concentration of 5 µM for the porphyrin and 4 µM for DPBF, and was then exposed to red light (*λ* = 647 nm) for 15 min (light dose, 9.63 J/cm^2^) at room temperature under constant stirring. The decrease of fluorescent intensity of the DPBF was recorded at 453 nm (*λ*_ex_ = 410 nm) before irradiation and after every 60 s of irradiation. From the obtained photodegradation curves, the area under the curve (AUC) was calculated according to the formula (Equation (S5)) previously described in Ref. [24], and the results are presented as an average of measurements in duplicate, with standard deviation in the error bars.

### 2.4. Lipophilicity

The lipophilicity of (oxidopyridyl)porphyrins and *N*-methylated pyridiniumporphyrins was calculated theoretically as a cLog*P* value, using the ALOGPS 2.1 program. Molecules were provided as SMILES.

### 2.5. Light Sources

The light sources used in this work were provided by the Centre of Excellence for Advanced Materials and Sensing Devices at the Ruđer Bošković Institute in Zagreb, and were described previously in Ref. [14]. A red-light source (*λ*_MAX_ = 647 nm, Δ*λ*_FWHM_ = 22 nm) with a fluence rate of 10.7 mW/cm^2^ was used for the measurement of ROS and singlet oxygen using the DPBF method, while a red-light source (*λ*_MAX_ = 643 nm, Δ*λ*_FWHM_ = 20 nm) with a fluence rate of 2 mW/cm^2^ and the uniform illumination of a standard 96-well plate was used for the in vitro studies.

### 2.6. In Vitro Studies

#### 2.6.1. Reagents and Chemicals

Cobalt(II) chloride, CoCl_2_ (#7646-79-9), was used for hypoxia induction and was purchased from Sigma-Aldrich (St. Louis, MO USA). Polyacrylamide gels were prepared by mixing 30% acrylamide solution (#1610156, Bio-Rad, Hercules, CA, USA), Tris-buffered saline, 10% sodium dodecyl sulphate (SDS) (#151-21-3, Fisher Scientific, Waltham, MA, USA), 10% ammonium persulfate (APS) (#7727-54-0, Fisher Scientific, Waltham, MA, USA) and N,N,N′,N′-tetramethylethane-1,2-diamine (TEMED) (#2367.3, Carl Roth, Karlsruhe, Germany). The recombinant rabbit monoclonal antibody used for HIF-1α detection in ChIC/CUT&RUN-seq (#ab51608) was purchased from Abcam (Cambridge, UK) and that for the detection of β-actin (horseradish peroxidase (HRP) conjugated, #A3854) was obtained from Sigma-Aldrich (St. Louis, MO, USA). The secondary anti-rabbit antibody labelled with HRP (anti-rabbit #111-035-144) was purchased from Jackson (West Grove, PA, USA). The nitrocellulose membrane was part of the Trans-Blot Turbo RTA Transfer Kit (#1704270) from Bio-Rad (Hercules, CA, USA), the protease and phosphatase inhibitor cocktails (#11873580001) used in the RIPA buffer were from Roche (Basel, Switzerland) and the Pierce ECL Western Blotting Substrate (#32209) was from Thermo Fisher Scientific (Waltham, MA, USA). In the fluorescence microscopy analysis, 3,3′-dihexyloxacarbocyanine iodide (DIOC_6_(3)) (#sc-205905) purchased from Santa Cruz Biotechnology (Santa Cruz, CA, USA) was used for labelling the mitochondria and endoplasmic reticulum (ER). The cytotoxicity of the porphyrins was evaluated with an MTT assay, using thiazolyl blue tetrazolium bromide (#M2128) purchased from Sigma-Aldrich (St. Louis, MO, USA).

#### 2.6.2. Cell Lines and Culturing Conditions

In this work, human malignant melanoma cell lines, amelanotic A375 and melanotic MeWo cells, and human dermal fibroblast (HDF) were used. Cell lines were cultured in Dulbecco’s Modified Eagle Medium (DMEM), supplemented with 10% FBS (Pan Biotech, Aidenbach, Germany), 1% L-glutamine solution (Pan Biotech, Aidenbach, Germany) and 1% penicillin-streptomycin solution (Pan Biotech, Aidenbach, Germany). Incubation was in a humidified atmosphere at 37 °C and 5% CO_2_, and the cells were passaged at 80–90% confluency.

#### 2.6.3. Hypoxia Induction with CoCl_2_ and Western Blot Analysis

The cells were seeded into 12-well plates at a concentration of 10^5^ cells/well. After the cells had reached full morphology (approx. 48 h), the medium was replaced with fresh medium containing cobalt(II) chloride (CoCl_2_) at a concentration of 100 μM, followed by incubation at 37 °C for 1 and 2 h. In the control sample, fresh medium without CoCl_2_ was added. After the incubation, cells were kept on ice and washed with ice-cold 1x PBS. After removing the 1x PBS, the cells were lysed with ice-cold radio-immunoprecipitation assay buffer (RIPA) buffer (50 mM Tris, 150 mM NaCl, 0.5% sodium deoxycholate, and 1% Triton X-100) and then incubated on ice for 30 min. The cells were centrifuged at 14,000 rpm at 4 °C for 10 min to separate the DNA in pellets and proteins in the supernatant. The supernatant was collected, and 4× Laemmli sample buffer (50 mM Tris pH 6.8, 10% glycerol, 2% SDS, 2% 2-mercaptoethanol, and 0.04% bromophenol blue) was added. The samples were heated at 95 °C for 10 min before separation on 10% polyacrylamide gels (SDS-PAGE). All gels were transferred to nitrocellulose membranes using the semi-dry transfer system at 100 V (Trans-Blot Turbo RTA Transfer kit, Bio Rad, Hercules, CA, USA, #1704270). The membranes were incubated for 1 h with a blocking buffer consisting of 3% bovine serum albumin (BSA) and 0.1% Tween 20 in 1× Tris-buffered saline, followed by immunoblotting with the primary antibody for HIF-1α overnight at 4 °C. After washing 3 times for 10 min with 0.1% Tween 20 in 1× Tris-buffered saline, the membranes were incubated at room temperature for 1 h with the secondary antibody, washed again according to the protocol described above and then developed with Pierce ECL Western blotting substrate, using the ChemiDoc Imaging System (Bio-Rad, Hercules, CA, USA). Densitometric analyses were performed using ImageJ software (version 1.54p, National Institutes of Health, Bethesda, MD, USA), and the HIF-1α protein levels were normalised to the loading control (β-actin).

#### 2.6.4. Cellular Uptake, Localisation and (Photo)Cytotoxicity Assays

Cellular uptake, intracellular localisation and (photo)cytotoxicity experiments were performed, following the procedures described in our previous work [14]. In the cellular uptake studies, the fluorescence was measured at *λ* = 650 nm and bandwidth 20 nm (λ_ex_ = 420 nm; bandwidth 9 nm) on the microplate reader, the Tecan Infinite 200Pro (Tecan Life Sciences, Zürich, Switzerland). All the measurements were performed in triplicate, and the obtained data is an average of repeated measurements, with standard deviation in error bars.

In the localisation tests, the cells were incubated with porphyrins and DIO_6_(3), a marker for mitochondria and endoplasmic reticulum (ER), which was used to investigate the colocalisation of porphyrins with the mentioned organelles. All images were analysed using the ImageJ program, while Pearson’s correlation coefficient was calculated using the CellSense program, fluorescence microscope software produced by Olympus.

For (photo)cytotoxicity assays, stock solutions of porphyrins were prepared in DMSO and dissolved in DMEM to the final concentrations. The porphyrin incubation time was 6h. Irradiation of the cells was with red light (*λ* = 643 nm) for 30 min (light dose, 3.6 J/cm^2^). Nonirradiated cells were used to test the dark toxicity. All measurements were performed in three individual experiments, and the results are shown as the proliferation (%) of the cells and were calculated as *IC*_50_ values.

### 2.7. Statistical Analysis

Statistical analysis was performed using GraphPad Prism 8 (GraphPad Software, Inc., San Diego, CA, USA) and the included standard two-way ANOVA test with post hoc Tukey analysis, at a confidence level of α = 0.05. The significance was set at *p* < 0.0001 and was shown using the following signs: **** *p* < 0.0001; *** *p* < 0.001; ** *p* < 0.01; * *p* < 0.1 and ns (not significant) > 0.1.

## 3. Results and Discussion

### 3.1. Synthesis of (Oxidopyridyl)Porphyrins

Selected *N*-methylated pyridiniumporphyrins from our previous studies [14] were prepared—one with only an acetamido group (**TMPyP3-CH_3_**), which we use in our studies as a hydrophilic PS (without a lipophilic chain), and three with different alkyl chain lengths (**TMPyP3-C_9_H_19_**, **TMPyP3-C_13_H_27_** and **TMPyP3-C_17_H_35_**) for lipophilicity comparison with the corresponding porphyrin precursors (**TPyP3-CH_3_**, **TPyP3-C_9_H_19_**, **TPyP3-C_13_H_27_** and **TPyP3-C_17_H_35_**). From the same precursors, *N*-oxide analogues were prepared (Figure 1), of which three were new compounds (**TOPyP3-CH_3_**, **TOPyP3-C_9_H_19_** and **TOPyP3-C_13_H_27_**), the structures of which have been confirmed by HRMS and ^1^H and ^13^C NMR spectroscopy (Figures S1–S6). The procedure for the *N*-oxidation [27] that we applied previously [22] to prepare **TOPyP3-C_17_H_35_** (confirmed by ^1^H NMR in Figure S7) has been modified here for all four (oxidopyridyl)porphyrins. The main difficulty in using *m*-CPBA as an oxidising reagent in excess is that triethylamine, which is used to stop the reaction to avoid the overoxidation of porphyrin (e.g., on pyrrole nitrogen atoms), yields triethylamine *N*-oxide upon oxidation, which is difficult to remove from the (oxidopyridyl)porphyrin product during purification. In a modified procedure that we applied here, it was shown that the subsequent removal of oxidised amine is easier if a more hydrophilic amine like propylamine (PrA) is used to quench the reaction. For all products, purification by column chromatography, with DCM and MeOH in different ratios as the eluent, had to be performed at least twice; for two compounds with longer alkyl chains (**TOPyP3-C_13_H_27_** and **TOPyP3-C_17_H_35_**), additional steps also had to be introduced before purification by column chromatography. In these additional steps, porphyrin was first protonated with an aqueous solution of strong acid (TFA) and, thus, dissolved in water, then 1M NaOH was added until precipitation. The precipitate was then extracted with dichloromethane, and the organic layer was washed twice with water. The final yields of (oxidopyridyl)porphyrin products ranged from 39 to 79%.

### 3.2. Spectroscopic Properties of (Oxidopyridyl)Porphyrins

**Figure 2:**
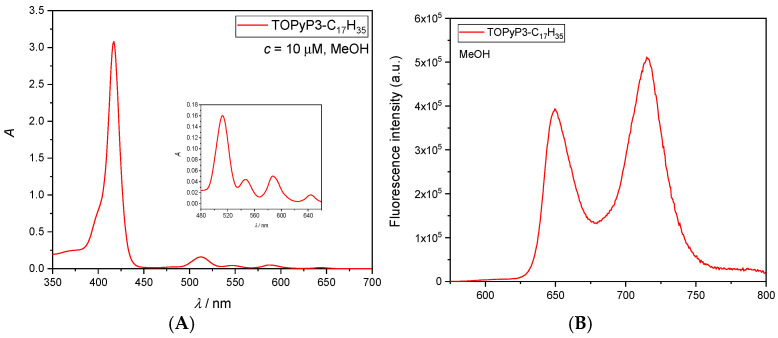
Absorption (**A**) and fluorescence (**B**) spectra of **TOPyP3-C_17_H_35_** in MeOH, representative of the (oxidopyridyl)porphyrins presented in this work.

It is known that *N*-oxides are stable in polar solvents [16,17]; therefore, our prepared (oxidopyridyl)porphyrins are also soluble in MeOH and are stable in this solution, owing to the formation of hydrogen bonds with the solvent molecules. Their absorption and fluorescence spectra were recorded in MeOH, and the spectra for **TOPyP3-C_17_H_35_** as a representative compound from this group are shown in Figure 2. In the absorption spectrum, a strong Soret band can be seen with a maximum at 417 nm, followed by four Q bands with maxima at 512, 547, 587 and 646 nm. In the fluorescence spectrum, two maxima are at 648 nm and 713 nm; thus, there is a small Stokes shift of 2 nm.

When comparing their absorption properties with their *N*-methylated analogues [14], *N*-oxide porphyrins show a bathochromic shift of the Soret band and higher values of the molar absorption coefficient (Table 1). Compared to the non-oxidised precursor, **TPyP3-C_17_H_35_** [22], **TOPyP3-C_17_H_35_** shows a very small hypsochromic shift in the Soret band and two Q_y_ bands, which is also representative of other (oxidopyridyl)porphyrins.

**Table 1:** Absorption and fluorescence properties of (oxidopyridyl)porphyrins.

	*λ*_abs_/nm (*ε/×* 10^3^ M^−1^cm^−1^)					*λ*_FL/_nm		*Φ*_FL_ *
	Soret (B)	Q_y_ (1-0)	Q_y_ (0-0)	Q_x_ (1-0)	Q_x_ (0-0)	Q(0,0)	Q(0,1)	
**TOPyP3-CH_3_**	416 (346.9)	510 (18.2)	545 (5.2)	588 (5.8)	645 (1.9)	648	713	0.050 ± 0.002
**TOPyP3-C_9_H_19_**	417 (296.4)	512 (15.4)	548 (5.1)	588 (4.9)	645 (1.5)	648	713	/
**TOPyP3-C_13_H_27_**	417 (364.6)	512 (19.3)	547 (5.8)	588 (6.0)	646 (2.0)	648	713	/
**TOPyP3-C_17_H_31_**	417 (275.4)	512 (15.0)	547 (4.3)	587 (4.5)	646 (1.6)	648	713	0.047 ± 0.002

* **TPP** was used as the reference compound; *Φ*_FL_= 0.11 [23].

The fluorescence quantum yield (*Φ*_FL_) for **TOPyP3-CH_3_** and **TOPyP3-C_17_H_35_**, the hydrophilic and lipophilic representatives of (oxidopyridyl)porphyrins, respectively, was determined using **TPP** as a standard (Table 1). The values of *Φ*_FL_ are very close, which indicates that substitution with fatty acid does not affect this photophysical property. Furthermore, the values of *Φ*_FL_ are somewhat lower compared to those of their *N*-methylated analogues, **TMPyP3-CH_3_** (*Φ*_FL_ = 0.072 [14]) and **TMPyP3-C_17_H_35_** (*Φ*_FL_ = 0.105 [24]).

#### Singlet Excited and Triplet Excited State Properties

Fluorescence decay measurements were performed using TC-SPC (Figure S8), and the lifetimes (*τ*_FL_) of **TOPyP3-CH_3_** and **TOPyP3-C_17_H_35_**, as obtained in MeOH, are presented in Table 2. As previously observed for their *N*-methyl analogues [14,24] and also for these two (oxidopyridyl)porphyrins, two decay times were determined: a short decay time (0.82 for **TOPyP3-CH_3_** and 0.57 ns for **TOPyP3-C_17_H_35_**) with a very small contribution (2% and 1%, respectively), and a longer decay time (8.62 and 7.82 ns) with a large contribution (98% and 99%). The very small contribution with short decay times suggests that porphyrins are mainly present in their monomeric form, and that the formation of aggregates in MeOH is not very likely, even with **TOPyP3-C_17_H_35_**, which has the longest alkyl chain.

The properties of the triplet excited state (^3^PS*) and the photophysical parameters for the porphyrins **TOPyP3-CH_3_** and **TOPyP3-C_17_H_35_** were determined by LFP, using **TPP** as the standard. In the transient absorption spectra of **TOPyP3-C_17_H_35_**, the absorption maxima were detected at 440 nm for the ^3^PS* and at 420 nm for the ground state bleach (Figure S9). The obtained lifetimes of the ^3^PS* (*τ*_T_) for **TOPyP3-CH_3_** (1.1 ms) and **TOPyP3-C_17_H_35_** (0.96 ms) are slightly longer in comparison to their *N*-methylated counterparts, **TMPyP3-CH_3_** (0.77 ms) and **TMPyP3-C_17_H_35_** (0.74 ms). The estimated quantum yield values of the intersystem crossing (*Φ*_ISC_) are higher for these two (oxidopyridyl)porphyrins (0.66 and 0.51, respectively, Table 2) compared to their *N*-methyl porphyrin analogues (0.34 and 0.38 [14,24]), indicating that better photophysical properties are needed in PSs for PDT.

### 3.3. ROS and Singlet Oxygen Production

**Figure 3:**
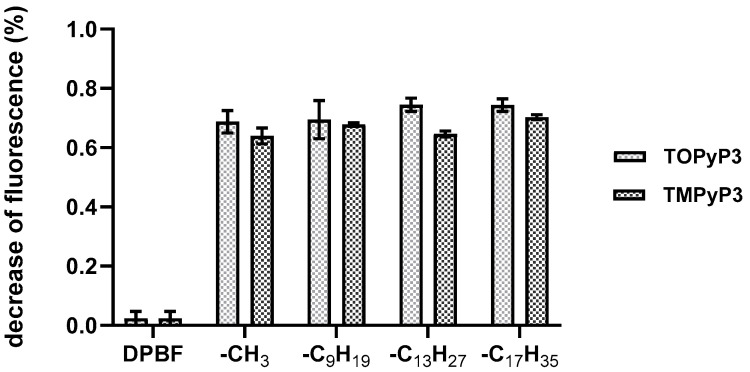
ROS/singlet oxygen production of the *N*-methylated pyridiniumporphyrins (**TMPyP3**) and (oxidopyridyl)porphyrins (**TOPyP3**) (5 µM), as measured by the photodegradation of DPBF (4 µM) in MeOH after irradiation with red light for 15 min (*λ* = 647 nm; 10.7 mW/cm^2^; total light dose 9.63 J/cm^2^).

For the ROS and ^1^O_2_ production measurements of four (oxidopyridyl)porphyrins, a relative method based on the photodegradation of DPBF was used, as in our previous work [14]. Their *N*-methylated analogues were evaluated under the same conditions for comparison. All (oxidopyridyl)porphyrins, regardless of their alkyl chain length, showed similar ROS/^1^O_2_ production, with more than 70% of DPBF fluorescence decreasing, and each of them showed somewhat higher production values compared to their respective *N*-methyl porphyrin analogue (Figure 3).

Taking into account the fact that all porphyrins in this experiment are soluble in methanol, without signs of aggregation, and that they had similar absorbance at the excitation wavelength (647 nm), it can be suggested that *N*-oxide porphyrins are slightly stronger producers of ROS/^1^O_2_, which coincides with their preferred photophysical properties (longer triplet excited state lifetimes and higher *Φ*_ISC_ values) compared to *N*-methylated ones.

### 3.4. Lipophilicity of (Oxidopyridyl)Porphyrins Compared to N-Methylated Pyridiniumporphyrins

In the development of new photosensitisers, as well as other therapeutics, it is necessary to consider the ADMET properties of the molecule at the earliest possible stage of research, and one of the most important physicochemical properties that plays an important role in this is lipophilicity. In our previous work, the lipophilicity of porphyrins (free-base and Zn(II)-chelated *N*-methylated pyridiniumporphyrins) was assessed experimentally using a modified shake-flask method, and the obtained Log*P* values were compared with those obtained theoretically using Chemicalize from ChemAxon [14]. In the case of the (oxidopyridyl)porphyrins in this work, we could not obtain data on lipophilicity using the shake-flask method, probably due to their zwitterionic nature; therefore, of the various free online tools available for calculating these values, we decided to use the ALOGPS 2.1 program (Table 3). This program was chosen because the theoretical values obtained after using it for porphyrins in our previous research are the closest to the experimentally determined values, while for the calculated values, we used other programs (such as SwissADME [28], Chemicalize [29] and miLog*P* from molinspiration.com) and observed very large deviations.

In our previous study, the lipophilic chain derived from the fatty acid was shown to be crucial in order for the porphyrin to enter the cell. *N*-methylated pyridiniumporphyrin, with the longest alkyl chain (**TMPyP3-C_17_H_35_**), showed the highest cell internalisation (HDF, MeWo and A375) after 24 h of incubation, especially at 37 °C, compared to the uptake at 4 °C [14]. The other porphyrins had an uptake proportional to the length of the alkyl chain, but **TMPyP3-C_17_H_35_** had a significantly larger uptake compared to the next in the series, **TMPyP3-C_15_H_31_**. Moreover, looking at the cellular uptake kinetics, all other porphyrins with shorter alkyl chains yielded the largest amount after 6 h of incubation, while for **TMPyP3-C_17_H_35,_** the concentration levels of the porphyrin inside the cell continued to increase with prolonged incubation.

**Figure 5:**
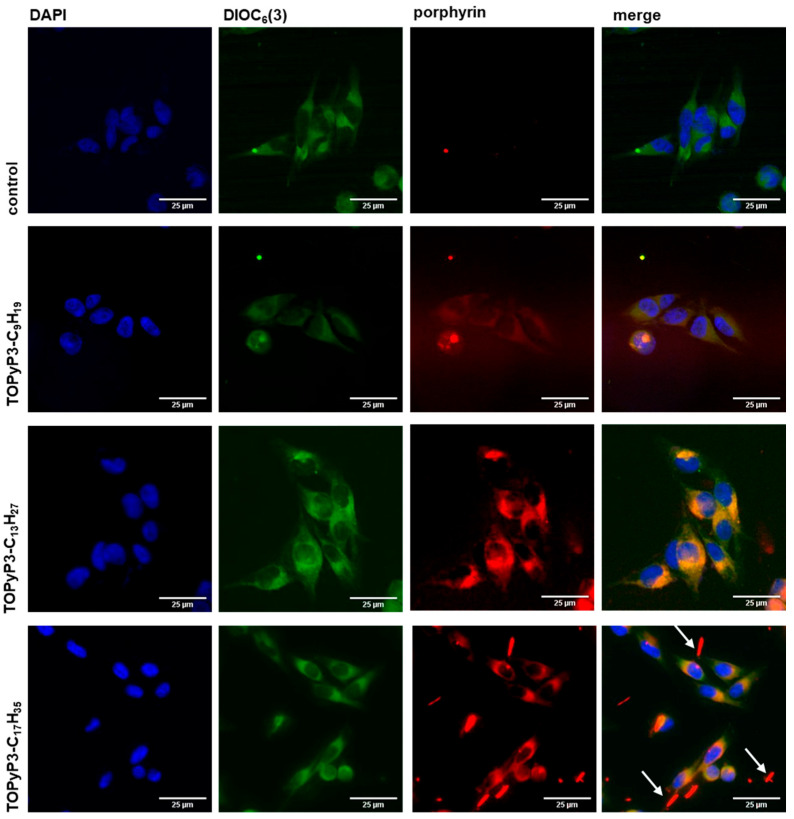
Localisation of the (oxidopyridyl)porphyrins with alkyl chains with 9 (**TOPyP3-C_9_H_19_**)*,* 13 (**TOPyP3-C_13_H_27_**) and 17 (**TOPyP3-C_17_H_35_**) C atoms, shown in MeWo cells incubated at a concentration of 5 µM for 6 h and with DIOC_3_(6) for 30 min. All images were taken by fluorescence microscopy at 20× magnification. The arrows are showing porphyrin aggregates outside the cells.

According to the experimentally obtained Log*P* values, it has been shown that **TMPyP3-C_17_H_35_** can be considered amphiphilic. Although the pyridyl-*N*-oxidation of (3-pyridyl)porphyrins increases the polarity of the molecule by introducing three *N*-oxide moieties, compared to cationic *N*-methylated pyridiniumporphyrins, zwitterionic (oxidopyridyl)porphyrins with the same length of the alkyl chain are, as expected, more lipophilic (Table 3). Lipophilicity with Log*P* values between 1 and 5, which applies to all (oxidopyridyl)porphyrins except for **TOPyP3-C_17_H_35_**, is considered adequate for membrane permeability, with a higher value within this range being desirable for higher-mass molecules. However, Log*P* values higher than 5 are associated with low solubility and a higher risk of in vivo toxicity [30]. In fact, **TOPyP3-C_17_H_35_** proved to be somewhat too lipophilic, and there were signs of aggregation of this PS in our localisation studies, showing porphyrin aggregates outside the cells (see Section 3.6. and the arrows in Figure 5).

**Table 3:** Calculated Log*P* values using the ALOGPS 2.1 program [31]. Structures were provided as SMILES.

(Oxidopyridyl)Porphyrins	cLog*P*	*N*-Methylated Porphyrins	cLog*P*
**TOPyP3-CH_3_**	2.52	**TMPyP3-CH_3_**	−1.60
**TOPyP3-C_9_H_19_**	4.08	**TMPyP3-C_9_H_19_**	−0.02
**TOPyP3-C_13_H_27_**	4.95	**TMPyP3-C_13_H_27_**	1.01
**TOPyP3-C_17_H_35_**	5.72	**TMPyP3-C_17_H_35_**	1.90

### 3.5. Cellular Uptake of (Oxidopyridyl)Porphyrins

**Figure 4:**
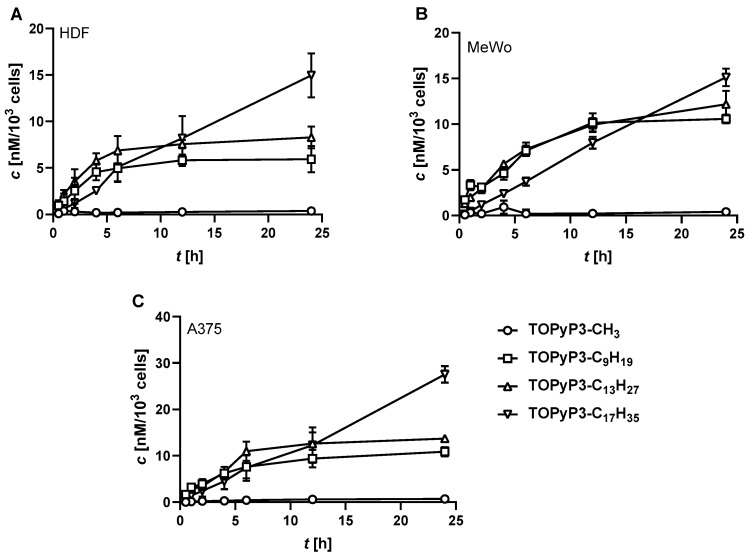
Cellular uptake kinetics, as measured over 24 h of incubation at 37 °C, of the (oxidopyridyl)porphyrins on the HDF (**A**), MeWo (**B**) and A375 (**C**) cell lines. The results are presented as a mean of calculated concentration per 10^3^ cells (nM), with the standard deviation in error bars obtained after three individual measurements.

The cellular uptake of (oxidopyridyl)porphyrins with an alkyl chain of different lengths was monitored by measuring the fluorescence at the Q(0,0) band wavelength of the cell lysates after incubation at 37 °C for up to 24 h, and the concentration of internalised porphyrin was calculated from the calibration curves (Figure S11). As can be seen in Figure 4, the porphyrin without an alkyl chain, **TOPyP3-CH_3_**, showed the lowest cellular uptake after 24 h of incubation compared to other (oxidopyridyl)porphyrins with an alkyl chain, and the amount of those porphyrins, as determined inside the cells, is proportional to their alkyl chain length. Interestingly, the uptake kinetics of (oxidopyridyl)porphyrins with an alkyl chain also varied, depending on the length of the alkyl chain. The porphyrins **TOPyP3-C_9_H_19_** and **TOPyP3-C_13_H_27_** showed similar kinetics, with the majority of the porphyrin being internalised within 6 h in the non-pigmented melanoma cell line (A375) and fibroblasts (HDF) and internalised within 12 h in the pigmented melanoma cell line (MeWo), in amounts that remained similar for up to 24 h. In contrast, **TOPyP3-C_17_H_35_**, porphyrins with the longest chains did not show saturation at any time-point tested, with a linear increase in uptake observed over 24 h. Although it is known that molecules of higher hydrophobicity are internalised much faster and/or in greater quantity compared to their more hydrophilic analogues [32], the results suggest that the porphyrin **TOPyP3-C_17_H_35_** with a Log*P* of > 5 may have too low a solubility, thereby affecting the kinetics of cellular uptake, which is much slower compared to other (oxidopyridyl)porphyrins substituted with shorter alkyl chains. This confirms the common assumption from the literature that the lipophilicity of porphyrins correlates with their cellular uptake [33,34], although their potential self-aggregation and interaction with membranes could affect their kinetics of internalisation [35].

Compared to the *N*-methylated porphyrin analogues with the same alkyl chain length (**TMPyP3-CH_3_**, **TMPyP3-C_9_H_19_**, **TMPyP3-C_13_H_27_** and **TMPyP3-C_17_H_35_**) [14], all (oxidopyridyl)porphyrin analogues exhibited lower intracellular concentration after 24 h of incubation, despite their higher lipophilicity, which probably led to the observed overall lower phototoxicity of (oxidopyridyl)porphyrins (Table 4).

In addition to the time-dependent cellular uptake, internalisation was measured after incubation at 4 °C or 37 °C for up to 24 h (Figure S12). As mentioned above, uptake at 37 °C correlates with the length of the alkyl chain, whereas at 4 °C, the highest uptake was observed for the porphyrin **TOPyP3-C_9_H_19_**, while the uptake for two other (oxidopyridyl)porphyrins with longer alkyl chains was lower. Therefore, the temperature-related difference in cellular uptake for **TOPyP3-CH_3_** and **TOPyP3-C_9_H_19_** was shown to be minimal, suggesting that passive uptake may be the predominant type of uptake. In the case of **TOPyP3-C_13_H_27_** and **TOPyP3-C_17_H_35_**, the temperature-induced difference in cellular uptake is significant, with the difference being greater for a porphyrin with a longer alkyl chain. Therefore, based on the uptake results and previous results, which indicated the aggregation of porphyrins substituted with a longer alkyl chain, it can be assumed that active transport is involved for porphyrins **TOPyP3-C_13_H_27_** and **TOPyP3-C_17_H_35_**, and that the longer the alkyl chain, the greater is the rate of active transport involved. These results are in correlation with those *N*-methylated analogues previously described, where it was also suggested that porphyrins with alkyl chains longer than 11 C atoms can achieve uptake by both passive and active mechanisms, with the ratio of active uptake being higher when the longer alkyl chain is substituted [14]. Further confirmation of the involvement of active transport is the potential formation of micelle-like aggregates [36] for porphyrins with a longer alkyl chain and binding to plasma proteins such as human serum albumin (HSA) through hydrophobic pockets [34], as observed with amphiphilic *N*-methylated analogues [14], which suppresses the passive pathway and favours endocytic uptake.

### 3.6. Localisation of (Oxidopyridyl)Porphyrins in MeWo Cells

In addition to efficient cellular uptake, the localisation of the PS has proven to be a decisive feature for the therapeutic success of PDT [32]. Mitochondria and the endoplasmic reticulum (ER) are organelles of interest as possible targets, since localisation in the mitochondria is known to trigger apoptosis as the main type of programmed cellular death, while localisation in the ER is known to activate both apoptosis and paraptosis [37,38].

To determine any potential preference for the ER and mitochondria, the localisation of (oxidopyridyl)porphyrins substituted with an alkyl chain of 9, 13 and 17 C atoms was observed by fluorescence microscopy incubation with 3,3′-dihexyloxacarbocyanine iodide (DIOC_6_(3)), a marker that is commonly used to target vesicle membranes, mitochondria and ER [39].

The results show that all the porphyrins tested are preferentially localised in the ER and/or mitochondria, which is evident by the yellow colouring of the merged images (Figure 5). In addition, the Pearson correlation coefficient was calculated using the ImageJ programme, and a value of > 0.90 for all three porphyrins was observed for localisation in the ER and mitochondria, while none of the porphyrins were found in the nucleus. In the case of **TOPyP3-C_17_H_35_**, the arrows indicate porphyrin that did not internalise in the cells and was not washed with PBS from the coverslips, proving its low solubility, as indicated by the calculated Log*P* value and the observed lower cellular uptake after 6 h of incubation. Although it seems that the observed colocalisation is high for all three porphyrins, the fluorescence microscopy images suffer from artefacts, which are usually a result of out-of-focus light. Therefore, we consider the obtained results with caution and will seek to confirm them through future experiments that should include confocal imaging and/or staining of the Golgi apparatus or the cytoskeleton.

### 3.7. (Photo)Cytotoxicity of (Oxidopyridyl)Porphyrins in Normoxia and CoCl_2_-Induced Hypoxia

The cytotoxicity of (oxidopyridyl)porphyrins was assessed by an MTT assay on the HDF, MeWo and A375 cell lines after 6 h of incubation with porphyrins, without irradiation, or after 30 min of irradiation with red light (*λ* = 643 nm), and under conditions of normoxia or hypoxia. To induce hypoxia, 100 µM of CoCl_2_ was added at least two hours before the porphyrins. Although this does not alter the concentration of oxygen, the replacement of iron(II) ions with cobalt(II) ions in prolyl hydroxylases blocks the hydroxylation of HIF-1α [40]. Oxygen is also one of the substrates for the activity of prolyl hydroxylases; therefore, in both cases, the result is the blockage of hydroxylation of HIF-1α and, consequently, its increase and stabilisation, which, in the case of CoCl_2_-induced hypoxia, can be maintained for several hours [40]. The accumulation of HIF-1α after treatment with 100 μM CoCl_2_ in DMEM after incubation for 1 and 2 h was confirmed by Western blotting on the A375 cell line (Figure S10).

**Figure 6:**
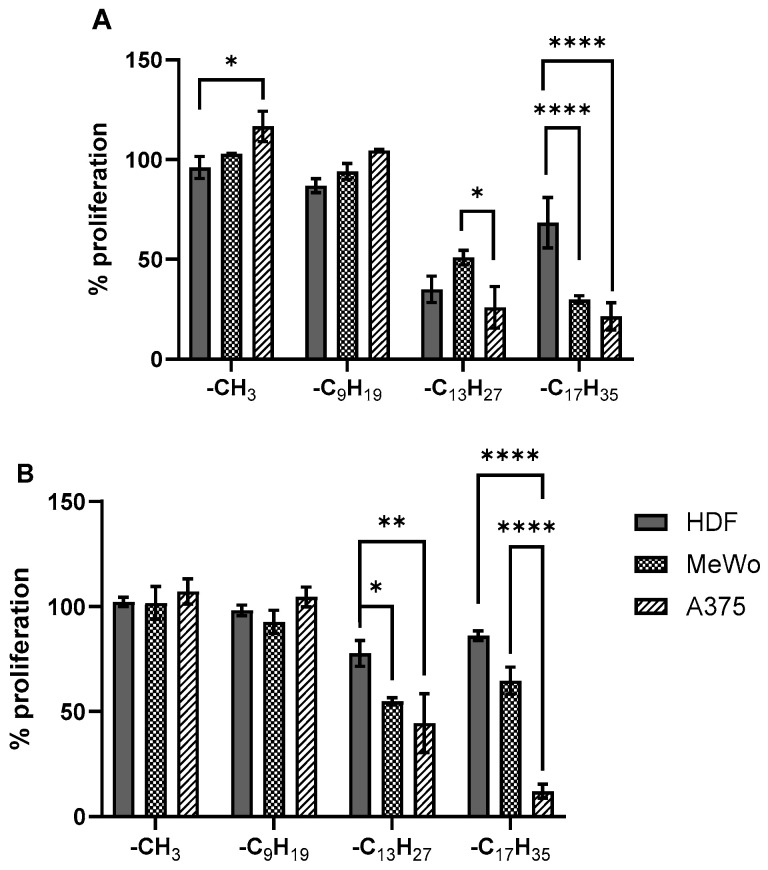
Comparison of the proliferation at a 1 µM concentration of (oxidopyridyl)porphyrins under the conditions of normoxia (**A**) and CoCl_2_-induced hypoxia (**B**) on the HDF, MeWo and A375 cell lines. The cells were irradiated at 643 nm. Statistical analysis was performed using a two-way ANOVA with a Tukey post hoc test (α = 0.05) to compare the toxicity of each porphyrin in different cellular lines. Significance was *p* < 0.0001 and was shown using the following signs: **** *p* < 0.0001; ** *p* < 0.01; * *p* < 0.1, ns (not significant) > 0.1.

None of the porphyrins showed ‘dark toxicity’ (i.e., without irradiation) on any cell lines and under both conditions in the concentrations tested (up to 100 µM). On the contrary, upon irradiation at 643 nm, when all four (oxidopyridyl)porphyrins were tested in concentrations of 1 µM, when under CoCl_2_-induced hypoxia, the proliferation of cells was similar or less reduced than under normoxia; however, this was shown to be statistically significant only with **TOPyP3-C_13_H_27_** on HDF and with **TOPyP3-C_17_H_35_** on MeWo (Figure S13). (Oxidopyridyl)porphyrin without an alkyl chain, **TOPyP3-CH_3_**, at a concentration of 1 μM did not appear to reduce the proliferation of any cell types, which can be related to the demonstrated absence of cell entry (Figure 4), while the phototoxicity of **TOPyP3-C_9_H_19_** was very low and was without significant difference in the different cells under conditions of either normoxia or hypoxia (Figure 6). Two (oxidopyridyl)porphyrins with longer alkyl chains showed a considerable reduction in the proliferation of cells in normoxia, at more than 50% on all cell lines, apart from **TOPyP3-C_17_H_35_** on HDF (~40%). The reduction in cell proliferation was somewhat lower with these two porphyrins in hypoxia, but interestingly, **TOPyP3-C_13_H_27_** reduced the proliferation of MeWo cells significantly more than HDF in hypoxia, while there was no significant difference between these two cell lines in normoxia. Under both conditions, and with both porphyrins, the highest reduction was shown in the proliferation of A375 cells (Figure 6).

The obtained *IC*_50_ values (Table 4) once again confirmed the importance of the alkyl chain and cellular uptake, as for both porphyrins without an alkyl chain; in all cases, the *IC*_50_ value was greater than 100 µM. Cationic porphyrins exhibited higher phototoxicity than their *N*-oxide analogues with the same alkyl chain, in all cells and under all conditions, which can be linked to their higher cellular uptake after 6 h, as demonstrated in our previous study [14]. With *N*-methylated porphyrins, there is a clear increase in phototoxicity with increasing chain length, which is consistent with the improved entry into cells, although the differences between **TMPyP3-C_13_H_27_** and **TMPyP3-C_17_H_35_** are relatively smaller than those between **TMPyP3-C_9_H_19_** and **TMPyP3-C_13_H_27_**, and in all cases, the phototoxicity is highest on amelanotic A375 cells. Furthermore, in all cases with *N*-methylated porphyrins, the phototoxicity decreased under CoCl_2_-induced hypoxia. Interestingly, the lowest *IC*_50_ values in Table 4 are identical for **TMPyP3-C_13_H_27_** and **TMPyP3-C_17_H_35_** on A375 under conditions of normoxia, and the values are also identical for these two PSs, albeit somewhat higher, under hypoxia. Given the very high sensitivity of this cell line, as shown in PDT, it is possible that the maximum effect is achieved with a chain length of 13 C-atoms, and increasing the lipophilicity of PSs does not contribute to a greater effect. This can also be confirmed by the fact that CoCl_2_-induced hypoxia does not significantly reduce the PDT effect for these two PSs, and their *IC*_50_ values are also identical under such ‘hypoxia’, because the same amount of oxygen is actually present in all these experiments. A very similar trend can be seen with **TOPyP3-C_13_H_27_** and **TOPyP3-C_17_H_35_** on A375 under normoxic conditions and CoCl_2_-induced hypoxia (Table 4). However, higher *IC*_50_ values, indicating the reduced phototoxicity of all *N*-methylated porphyrins under CoCl_2_-induced hypoxia conditions compared with normoxia, are consistent with the literature, where it has been shown that accumulated HIF-1α enhances cell resistance to PDT [41]. Accumulated HIF-1α has been shown to promote tumour angiogenesis through the overexpression of vascular endothelial growth factor (VEGF) and to enhance tumour proliferation and survival through the overexpression of p53 [41,42]. In addition, it has been shown that ROS can be generated in hypoxia and, together with other free radicals in the TME, can directly or indirectly promote the stabilisation and activation of HIF-1α and its translocation to the nucleus by the cytokines [43]. Another mechanism of hypoxia-induced PDT resistance was proposed by Ji et al., where it was found that the stabilisation of HIF-1α, induced by the addition of CoCl_2_, resulted in decreased activity of 5-aminolevulinic acid (5-ALA)-mediated PDT on Het-1A (a non-cancerous endothelial cell line), which was partly attributed to the inhibition of apoptotic cellular death by PDT [44]. Furthermore, Rodriguez et al. showed that the resistance of PDT with 5-aminolevulinic acid methyl ester (Me-ALA) was due to the induction of autophagy as a survival mechanism [45]. The accumulation of HIF-1α, which is a consequence of ROS production by PDT and CoCl_2_, leads to the overexpression of vacuolar membrane protein 1 (VMP-1), a protein that plays an important role in the formation of autophagosomes [45]. The provided information suggests that the decreased photosensitisation seen in CoCl_2_-treated cells after treatment with both porphyrin series is the result of an induced accumulation of HIF-1α, which increases their resistance to ROS, cell survival and proliferation, as well as the inhibition of one of the major mechanisms of cellular death, apoptosis.

Unlike the *N*-methylated porphyrins, which, in all cases, have higher *IC*_50_ values in hypoxia, interesting deviations can be noted with the *N*-oxides. For the *N*-oxide with the longest chain, **TOPyP3-C_17_H_35_**, the *IC*_50_ value for HDF is lower in hypoxia than in normoxia, while the values for melanotic melanoma cells, MeWo, are almost identical in both conditions; even more interesting, those values are lower than for A375 (Table 4). This prompted us to compare their selectivity in phototoxicity on different melanoma cells (melanotic and amelanotic) between two groups of PSs under both normoxic and hypoxic conditions. The different selectivity of the drugs on differently pigmented melanoma cells has already been observed; for example, amelanotic (NM78-AM) and melanotic (NM78-MM) cell lines from human oral melanoma showed different sensitivities to different cancer drugs, while NM78-MM cells were shown to be sensitive to cisplatin, although NM78-AM were not, and so on [46]. For many PSs, melanin interferes with the absorption of light, and, thus, reduces their effectiveness in PDT, but it was also observed that the presence of melanin affects the type of cell death in PDT; for example, 5-aminolevulinic acid (5-ALA) PDT induced apoptosis in pigmented BF16 and autophagy in amelanotic B16 cell melanoma cells [47]. In addition to the competition for light, the complex antioxidant role of melanin in the protection of melanoma cells is also known, which generally leads to reduced effectiveness and increased resistance to PDT [48]. This can be clearly seen with our *N*-methylated PSs, for which the therapeutic index of selectivity (SI) is much higher for A375 (e.g., 10.6 for **TMPyP3-C_9_H_19_** in normoxia) than that for MeWo (2.0 for **TMPyP3-C_9_H_19_** in normoxia), although the selectivity in both cell lines decreases with the increasing lipophilicity of PSs, as does the difference between SI for A375 and MeWo (e.g., SI values are 5.4 and 1.9, respectively, for **TMPyP3-C_13_H_27_** and 2.2 and 1.0 for **TMPyP3-C_17_H_35_** in normoxia). These trends are very similar in normoxia and hypoxia; just the SI values are slightly lower in hypoxia or are almost identical for **TMPyP3-C_17_H_35_** (Table S1). In contrast, its *N*-oxide analogue, **TOPyP3-C_17_H_35_**, has slightly higher selectivity for MeWo than for A375 cells, both in normoxia (2.0 and 1.7, respectively) and in hypoxia (1.6 and 1.2). The other two (oxidopyridyl)porphyrins with a shorter alkyl chain, **TOPyP3-C_9_H_19_** and **TOPyP3-C_13_H_27_**, have low and very similar SI values for both MeWo and A375, but interestingly, their selectivity is somewhat higher in hypoxia. Furthermore, even though SI values for these two porphyrins are higher for A375 than for MeWo, these differences are much smaller than for their *N*-methylated analogues (Table S1).

The (oxidopyridyl)porphyrins prepared in this work did not show additional cytotoxic effects under CoCl_2_-induced hypoxia, which we aimed to achieve by introducing *N*-oxide functionalities as possible HAP moieties. However, it is important to emphasise that in this study, all (photo)cytotoxicity assays were conducted under the same conditions regarding oxygen concentration, and only chemical hypoxia induced by cobalt(II) chloride was used, which leads to the accumulation of HIF-1α. Considering that all *N*-methylated PSs with an alkyl chain had lower activity in such hypoxia conditions, which is consistent with the increase in resistance to PDT due to HIF-1α accumulation described in the literature, their *N*-oxide analogues showed interesting deviations, suggesting additional activity. Namely, although their recorded phototoxicity is lower than that of *N*-methylated porphyrins, which is most likely related to lower cell entry, the (oxidopyridyl)porphyrin with the longest chain, **TOPyP3-C_17_H_35_**, showed equal activity on MeWo under normoxia and hypoxia conditions, while on HDF, the activity in hypoxia was even somewhat higher. It is even more striking that (oxidopyridyl)porphyrins with an alkyl chain showed a smaller difference in selectivity between pigmented (MeWo) and non-pigmented (A375) melanoma cells; moreover, **TOPyP3-C_17_H_35_** demonstrated slightly greater selectivity for MeWo cells compared to A375, both in normoxia and in hypoxia (Table S1). Therefore, it seems that *N*-oxide porphyrins have possibly reduced the protective (antioxidant) role of melanin, perhaps by producing more ROS through an additional mechanism. These possibilities remain to be explored in future studies, and we plan to further investigate the activity of (oxidopyridyl)porphyrins in PDT under conditions of reduced oxygen concentration, i.e., physiological hypoxia, in comparison to chemical hypoxia.

## 4. Conclusions

The porphyrins that showed the highest PDT effect on melanoma cells (amelanotic A375 and melanotic MeWo) and fibroblasts (HDF) in this study were the two *N*-methylated pyridiniumporphyrins with the longest alkyl chains (**TMPyP3-C_13_H_27_** and **TMPyP3-C_17_H_35_**) and one (oxidopyridyl)porphyrin, the one with the longest alkyl chain (**TOPyP3-C_17_H_35_**). Their *IC*_50_ values were equal to or lower than 1 µM in all the tested cells, regardless of whether the cells were treated with CoCl_2_ or not. Both groups of porphyrins tested in this study have proven to be very effective producers of singlet oxygen and ROS, which is certainly beneficial for the treatment of pigmented melanoma; however, the strongest PDT effect on all cells was shown by porphyrins with the most effective entry into the cells. This is confirmed by the fact that *N*-methylated porphyrins, which were more efficient in terms of cellular entry, exhibited a stronger effect on all cells than their *N*-oxidised analogues, which had slightly higher ^1^O_2_/ROS levels. In contrast, unlike *N*-methylated porphyrins, *N*-oxides show a much smaller difference in selectivity between pigmented and non-pigmented melanoma cells, and the most potent of them, **TOPyP3-C_17_H_35_**, showed even greater selectivity on MeWo compared to A375 cells. Moreover, this (oxidopyridyl)porphyrin was equally effective on MeWo cells in normoxia and CoCl_2_-induced hypoxia. These results suggest that porphyrins with *N*-oxide moieties may have additional mechanisms for photocytotoxicity that oppose the protective, antioxidant role of melanin, along with conditions in the cells created by accumulated HIF-1α, but these mechanisms need to be investigated in future research.

## Figures and Tables

**Table 2:** Singlet and triplet excited state properties for porphyrins **TOPyP3-CH_3_** and **TOPyP3-C_17_H_35_**, with **TPP** as the reference compound obtained by time-correlated single-photon counting (TC-SPC)—lifetime of the singlet excited state (*τ*_S_)—and by laser flash photolysis (LFP)—lifetime of the triplet excited state (*τ*_T_), the quantum yield of the intersystem crossing (*Φ*_ISC_), the molar absorption coefficient of the triplet state (*ε*_T-T_), and the quenching rate constant of the triplet excited state by O_2_ (*k*_q_ (O_2_)).

Compounds	*τ*_FL_(% of the Decay)/ns	*τ*_T_/μs	ε_T-T_/M^−1^cm^−1^	*Φ*_ISC_	*k*q (O_2_)/M^−1^s^−1^
TPP *	10.7 ns	90	66 600	0.67	/
**TOPyP3-CH_3_**	*τ*_1_ = 0.82 ± 0.08 ns (2%) *τ*_2_ = 8.62 ± 0.02 ns (98%)	1100 ± 200	47 000	0.66	1.3 × 10^9^
**TOPyP3-C_17_H_35_**	*τ*_1_ = 0.57 ± 0.08 ns (1%) *τ*_2_ = 7.82 ± 0.02 ns (99%)	960 ± 30	58 300	0.51	1.4 × 10^9^

* **TPP** was used as a reference compound [23]. ns means not significant.

**Table 4:** Calculated *IC*_50_ values for *N*-methylated pyridiniumporphyrins (**TMPyP3**) and (oxidopyridyl)porphyrins (**TOPyP3**), conjugated with an alkyl chain of 1, 9, 13 and 17 C atoms, after irradiation with red light (*λ* = 643 nm, 2 mW/cm^2^, total light dose 3.6 J/cm^2^) for 30 min on the HDF, MeWo and A375 cell lines. Stock solutions were prepared in DMSO before being diluted in DMEM. The results are presented as an average value of the measurements in triplicate with standard deviation.

		HDF		MeWo		A375	
	*IC*_50_ [µM]	No CoCl_2_	CoCl_2_ *	No CoCl_2_	CoCl_2_ *	No CoCl_2_	CoCl_2_ *
**TMPyP3**	**-CH_3_**	>100	>100	>100	>100	>100	>100
	**-C_9_H_19_**	7.09 ± 0.66	8.87 ± 0.02	3.52 ± 0.65	6.81 ± 1.36	0.67 ± 0.03	0.99 ± 0.13
	**-C_13_H_27_**	0.81 ± 0.05	0.99 ± 0.05	0.43 ± 0.03	0.61 ± 0.16	0.15 ± 0.06	0.22 ± 0.08
	**-C_17_H_35_**	0.33 ± 0.03	0.46 ± 0.14	0.33 ± 0.04	0.47 ± 0.02	0.15 ± 0.08	0.23 ± 0.07
**TOPyP3**	**-CH_3_**	>100	>100	>100	>100	>100	>100
	**-C_9_H_19_**	3.49 ± 0.45	7.65 ± 0.06	5.46 ± 1.08	6.28 ± 0.82	3.87 ± 0.45	5.46 ± 1.48
	**-C_13_H_27_**	0.86 ± 0.04	2.62 ± 0.19	1.04 ± 0.09	1.79 ± 0.37	0.78 ± 0.17	0.86 ± 0.06
	**-C_17_H_35_**	1.31 ± 0.57	1.09 ± 0.29	0.66 ± 0.14	0.68 ± 0.19	0.77 ± 0.08	0.94 ± 0.15

* CoCl_2_ was added in a 100 μM concentration a minimum of 2 h before the porphyrin was added.

## Data Availability

Data is available in the Dabar Repository of the Faculty of Biotechnology and Drug Development, University of Rijeka (https://urn.nsk.hr/urn:nbn:hr:193:072251, accessed on 20 May 2025).

## Supplementary Materials

The following supporting information can be downloaded at: https://www.mdpi.com/article/10.3390/antiox14080992/s1, Figures S1–S7: ^1^H and ^13^C NMR spectra of (oxidopyridyl)porphyrins; Figure S8: Fluorescence decay of porphyrins TOPyP3-CH_3_ and TOPyP3-C_17_H_35_; Figure S9: Transient absorption spectra after 355 nm of laser excitation of TOPyP3-C_17_H_35_; Figure S10: HIF-1α protein levels after incubation with 100 μM CoCl_2_, a hypoxia mimetic agent; Figure S11: Calibration curves in 1% SDS in 0.1 M NaOH, as used for calculation of concentration of (oxidopyridyl)porphyrins in experiments on cellular uptake; Figure S12: Comparison of the cellular uptake at 4 °C and 37 °C after incubation for 24 h with (oxidopyridyl)porphyrins in the HDF (A), MeWo (B) and A375 (C) cell lines; Figure S13: Comparison of the proliferation at 1 µM concentration of (oxidopyridyl)porphyrins under conditions of normoxia and CoCl_2_-induced hypoxia on the HDF (A), MeWo (B) and A375 (C) cell lines; Table S1: Calculated selectivity index (SI) of melanoma cell lines, determined by dividing the obtained *IC*_50_ values for fibroblasts (HDF) with the *IC*_50_ values obtained for melanoma cells; Equation (S1): Fluorescence quantum yield (*Φ*_FL_); Equation (S2): Molar absorption coefficients for the triplet states (*ε*_T_); Equation (S3): Quantum yield of intersystem crossing (*Φ*_ISC_); Equation (S4): Quenching rate constant of a triplet excited state (*k*_q_); Equation (S5): Area under the curve (AUC) of the photodegradation (fluorescence decrease) of DPBF calculation.
