# Supplementary material for: (Oxidopyridyl)Porphyrins of Different Lipophilicity: Photophysical Properties, ROS Production and Phototoxicity on Melanoma Cells Under CoCl2-Induced Hypoxia
Source: Antioxidants (Basel). 2025 Aug 13;14(8):992. doi: 10.3390/antiox14080992 (PMC12382614; doi:10.3390/antiox14080992)
Supplement: Supplementary file 1 [file antioxidants-14-00992-s001.zip › antioxidants-3700014-Supplementary.pdf]

Supplementary information

for

(Oxidopyridyl)porphyrins of Different Lipophilicity: Photophysical properties, ROS Production and Phototoxicity on Melanoma Cells under $CoCl_2$-induced Hypoxia

Martina Mušković [1], Martin Lončarić [2], Ivana Ratkaj [1*], and Nela Malatesti [1,*]

[1] Faculty of Biotechnology and Drug Development, University of Rijeka, Radmile Matejčić 2, Rijeka 51000, Croatia; e-mail: martina.muskovic@biotech.uniri.hr

[2] Laboratory for Photonics and Quantum Optics, Division of Experimental Physics, Ruđer Bošković Institute, Bijenička cesta 54, Zagreb 10000, Croatia; martin.loncaric@irb.hr

*Correspondence: iratkaj@biotech.uniri.hr (I.R.), nela.malatesti@biotech.uniri.hr (N.M.)

Content:

1. ^{1}H and ^{13}C NMR spectra of synthesised (oxidopyridyl)porphyrins pg. 1

2. TC-SPC and LFP of porphyrins pg. 8
   2.1. Equations used to determine Φ_{FL}, Φ_{ISC} and AUC for DPBF photodegradation
   2.2. TC-SPC and LFP spectra for porphyrinsTOPyP3-CH_3 and TOPyP3-$C_{17}H_{35}$

3. Western blot analysis for HIF-1α stabilisation pg. 10
4. Cellular uptake of (oxidopyridyl)porphyrins (calibration curves and temperature-dependent cellular uptake pg. 11

5. (Photo)cytotoxicity of (oxidopyridyl)porphyrins on different cell lines pg. 13

1.^{1}H and ^{13}C NMR spectra of synthesised (oxidopyridyl)porphyrins

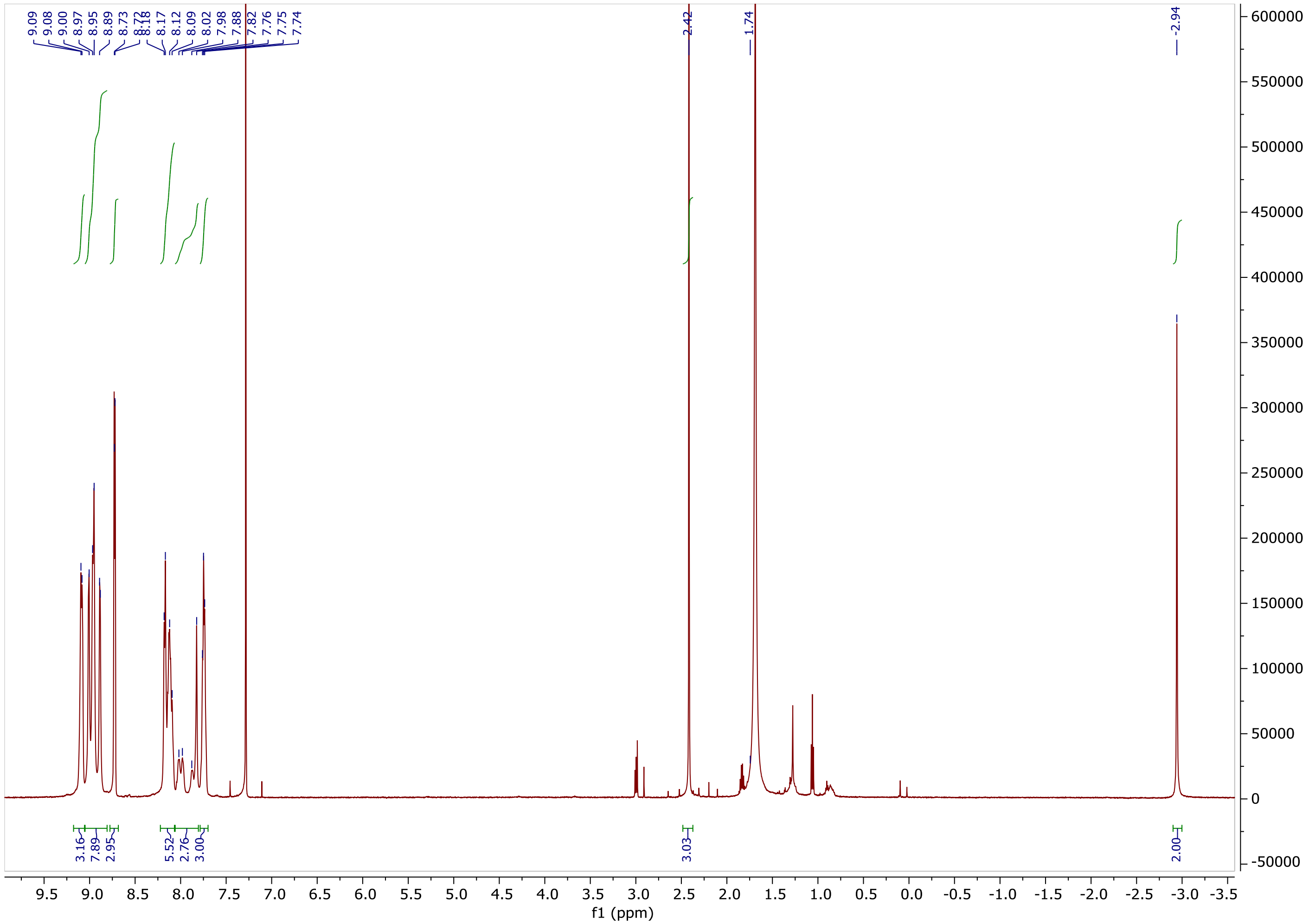

Figure S1. ^{1}H NMR ($CDCl_3$, 600 MHz) spectrum of porphyrin **TOPyP3-CH_3**.

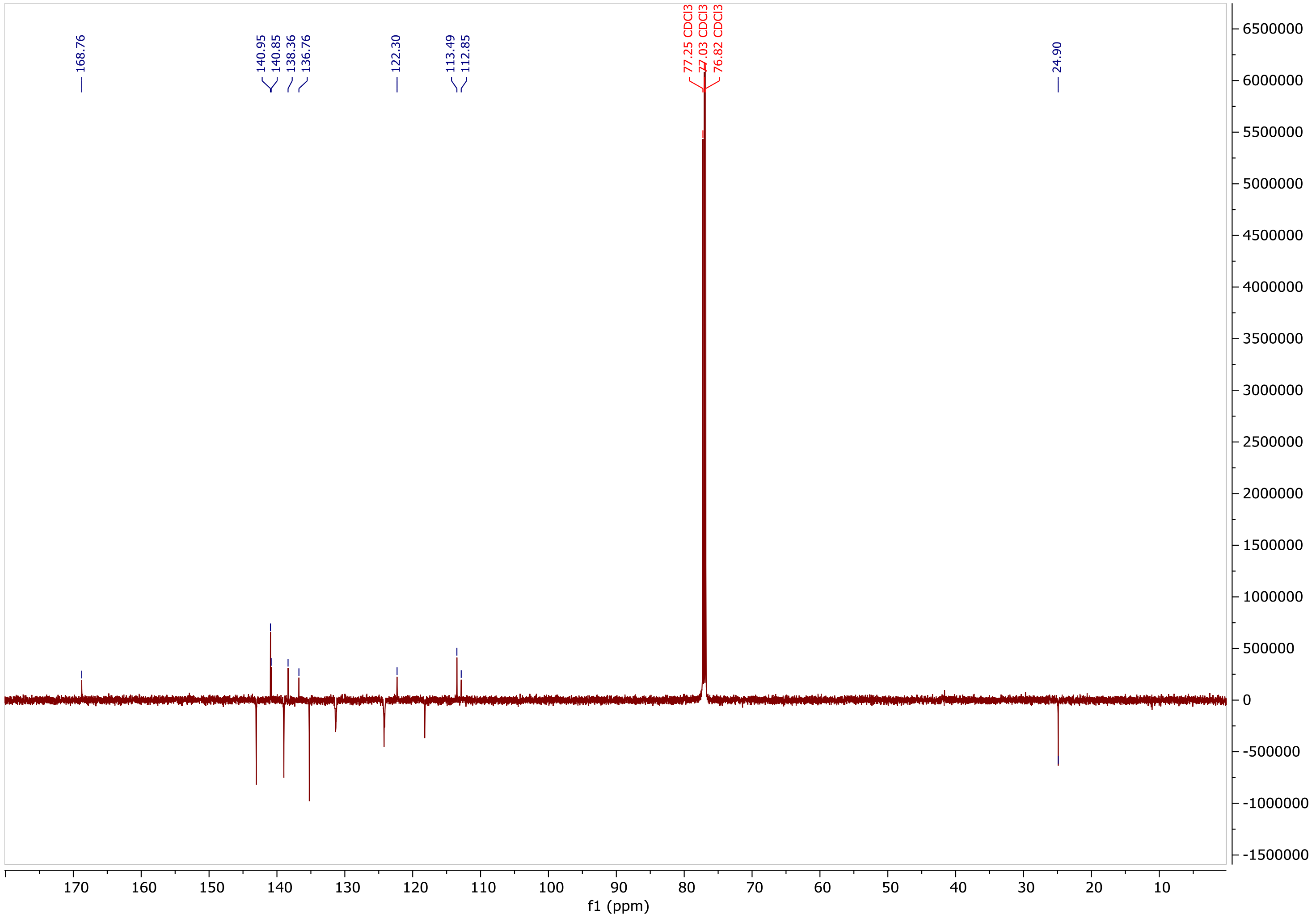

Figure S2. ^{13}C NMR ($CDCl_3$, 150 MHz) spectrum of porphyrin **TOPyP3-CH$_3$**.

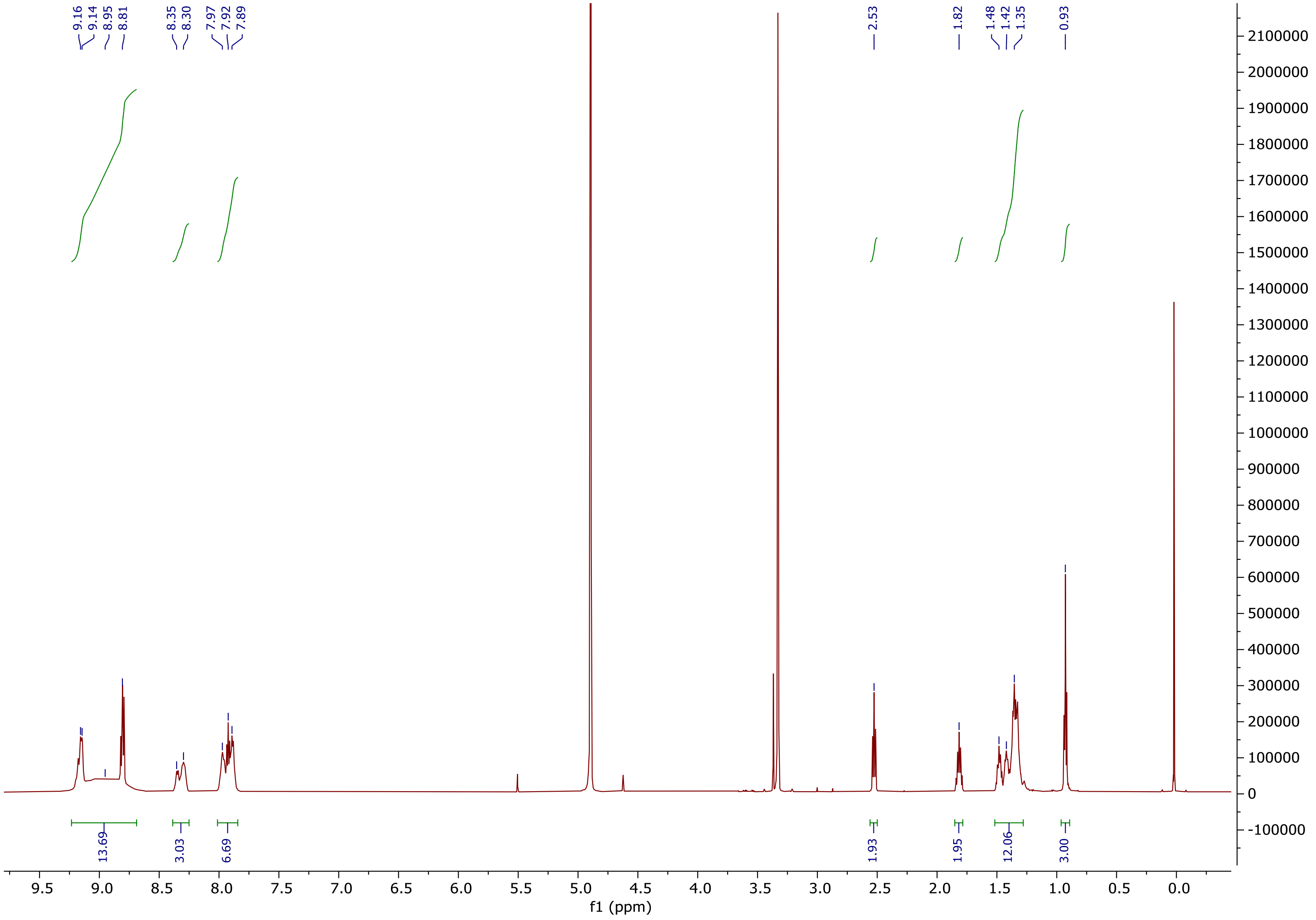

Figure S3. ^{1}H NMR (CD_3OD, 600 MHz) spectrum of porphyrin **TOPyP3-C_9H_{19}**.

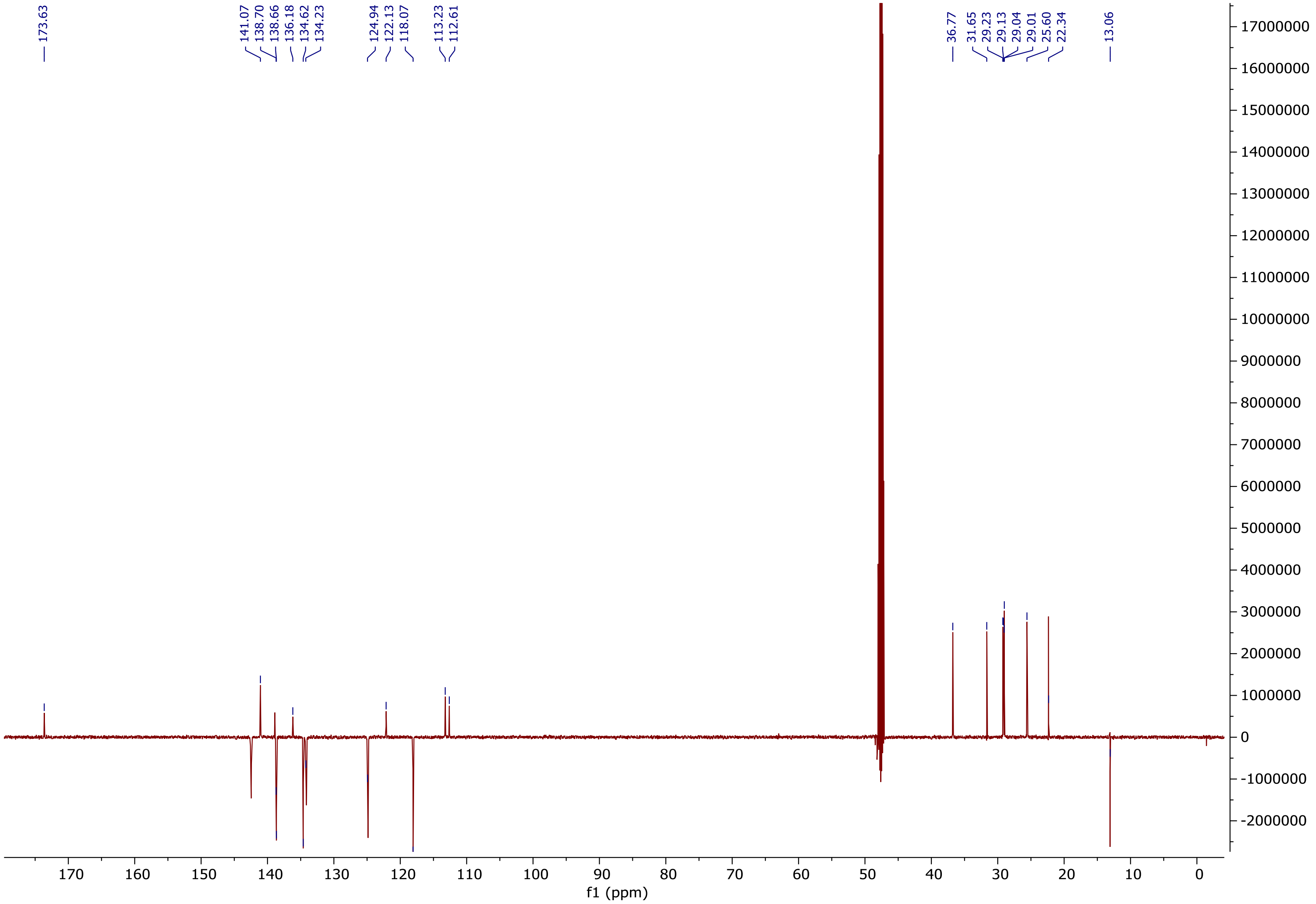

Figure S4. ^{13}C NMR (CD_3OD, 150 MHz) spectrum of porphyrin **TOPyP3-C$_9$H$_{19}$**.

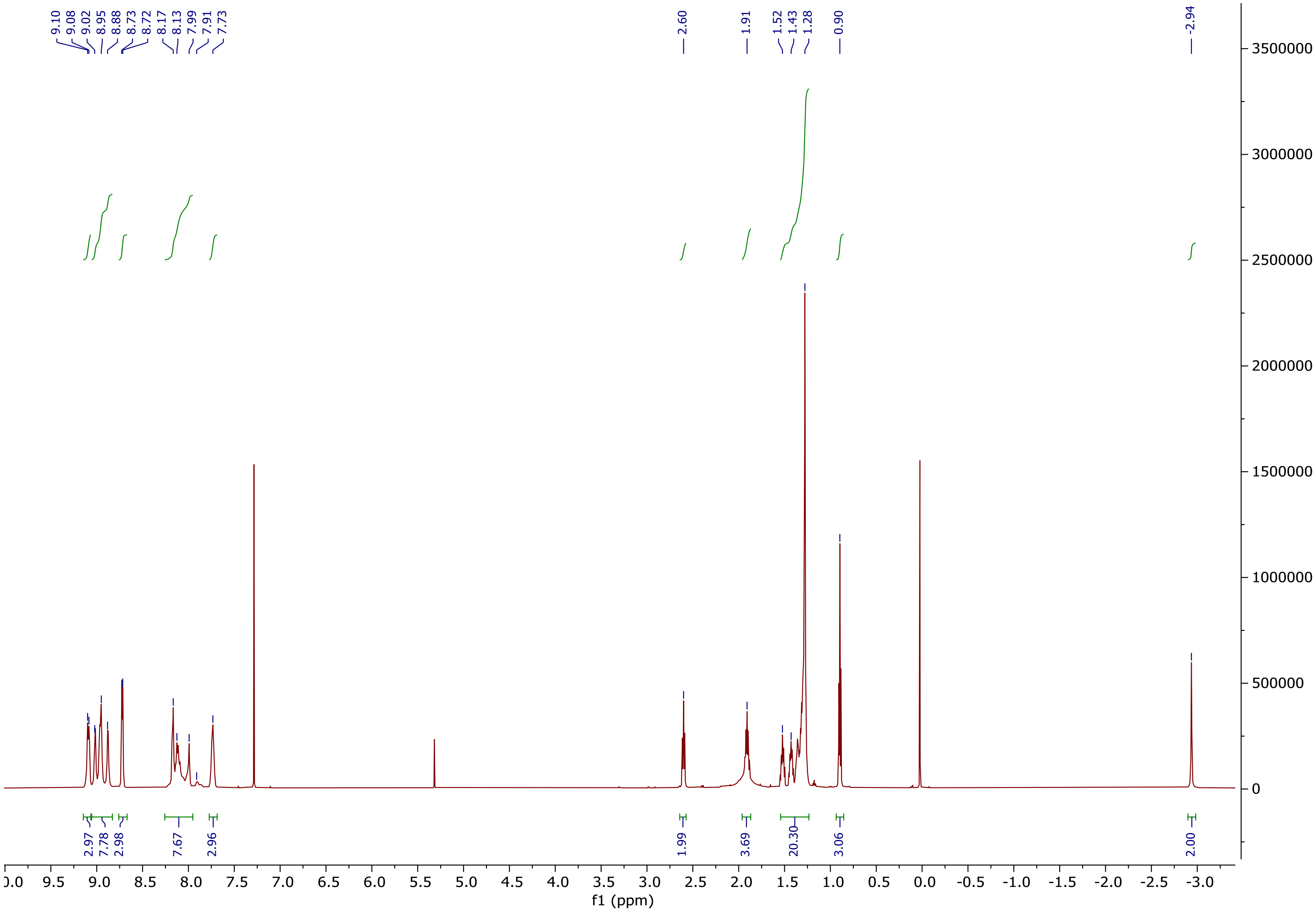

Figure S5. ^{1}H NMR ($CDCl_3$, 600 MHz) spectrum of porphyrin **TOPyP3-$C_{13}H_{27}$**.

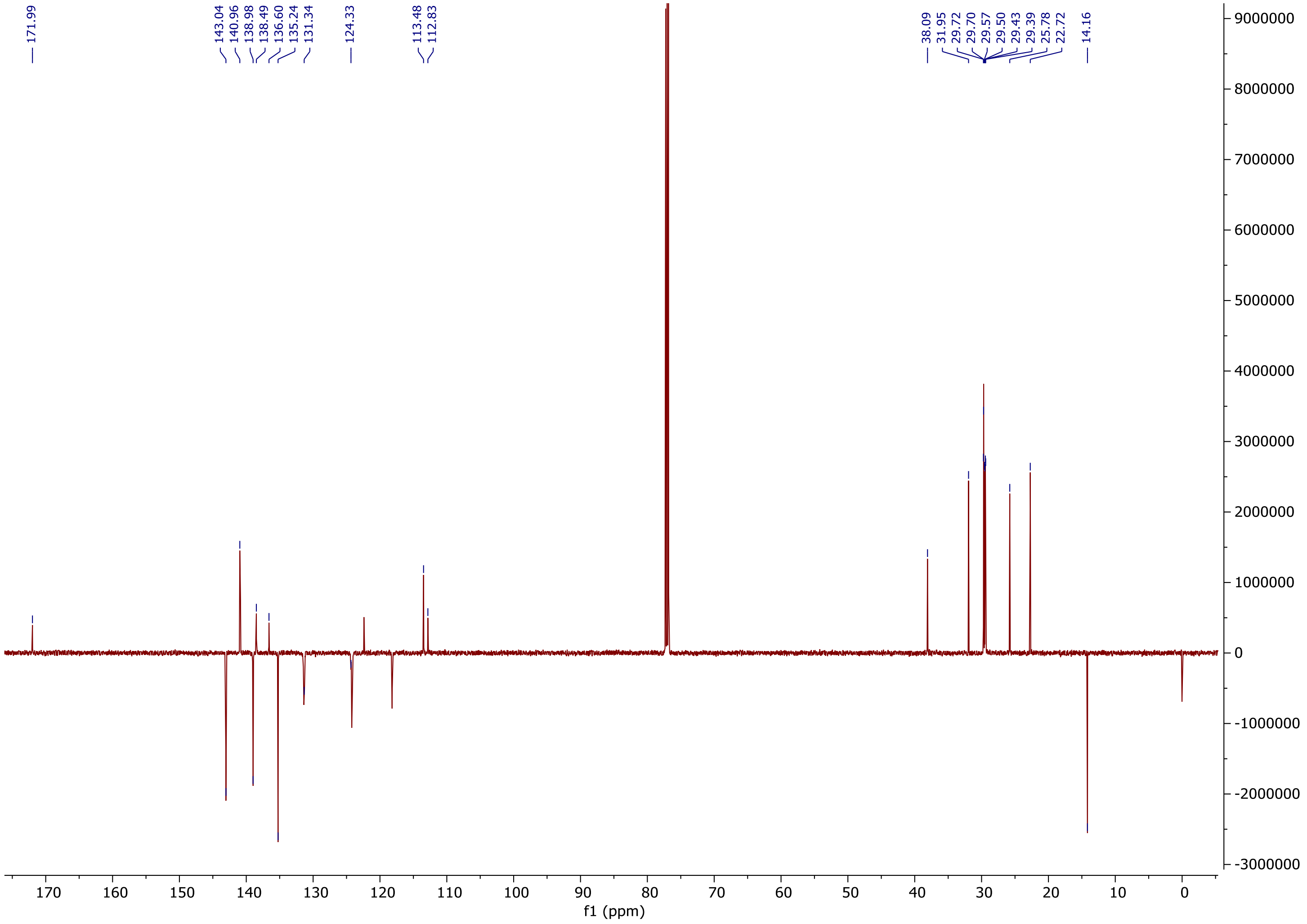

Figure S6. ^{13}C NMR ($CDCl_3$, 150 MHz) spectrum of porphyrin **TOPyP3-$C_{13}H_{27}$**.

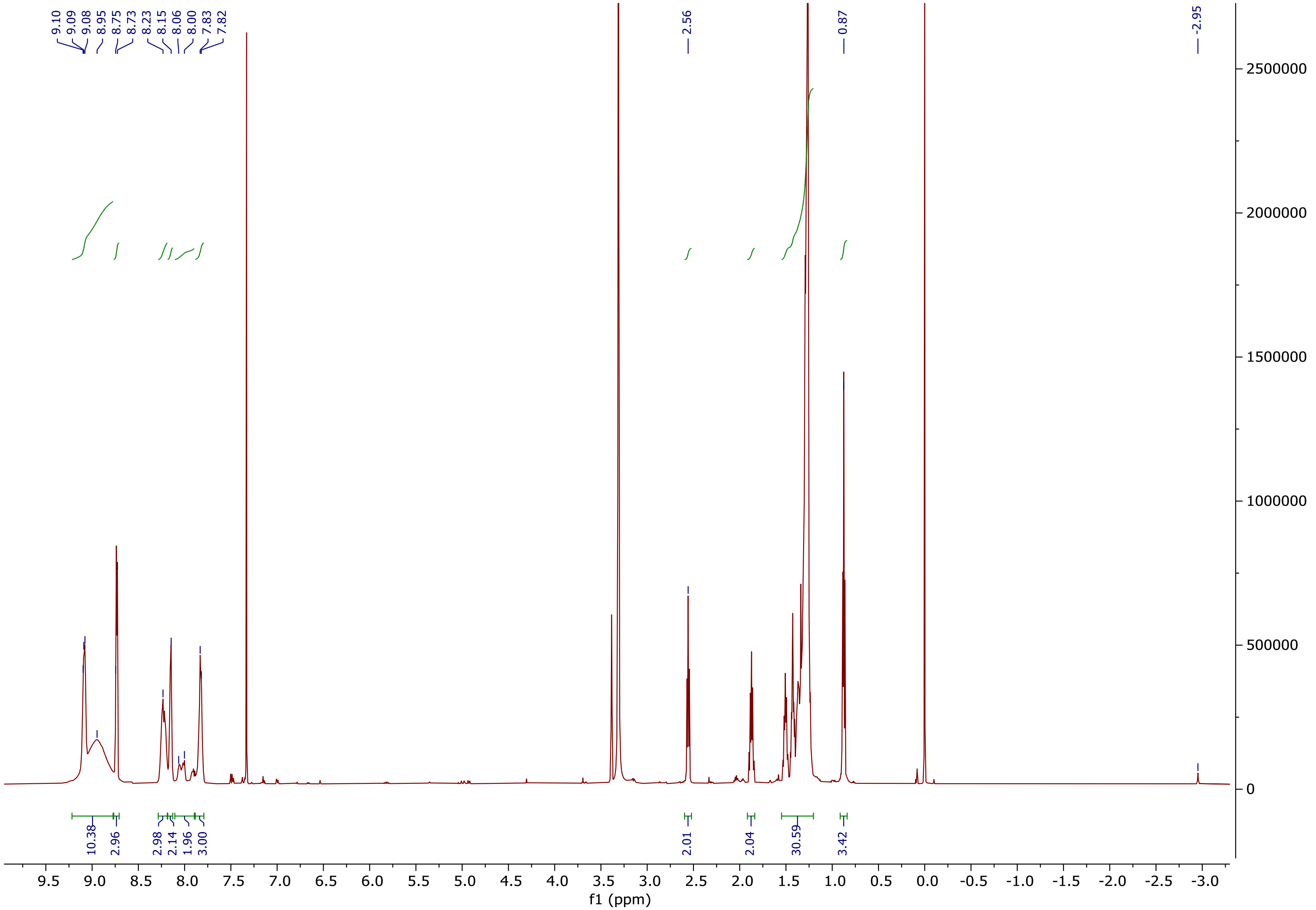

Figure S7. ^{1}H NMR ($CDCl_3$ + CD_3OD, 600 MHz) spectrum of porphyrin **TOPyP3-$C_{17}H_{35}$**.

2. TC-SPC and LFP of porphyrins

2.1. Equations used to determine Φ_{FL}, Φ_{ISC} and AUC for DPBF photodegradation

Fluorescence quantum yield (Φ_{FL}) was calculated according to:

$$\Phi_{FL} = \Phi_{FL(R)} \left(\frac{n}{n_R}\right)^2 \frac{I}{I_R} \frac{1-10^{-A_R}}{1-10^{-A}} \quad \text{(S1)}$$

Φ_{FL} and $\Phi_{FL(R)}$ – fluorescence quantum yield of the compound and the reference;

n and n_R – refractive index of the solvent in which compound or the reference was dissolved;

A and A_R – absorbance of the compound and the reference at the excitation wavelength;

I and I_R – area under emission curve of the compound and the reference.

Molar absorption coefficients for the triplet states (ε_T) were calculated using the singlet depletion method following the equation:

$$\varepsilon_T = \varepsilon_S \frac{\Delta A_T}{\Delta A_S} \quad \text{(S2)}$$

ε_T – molar absorption coefficient of the triplet state

ε_S – molar absorption coefficient of the ground state

ΔA_T – changes in the absorption at the triplet maximum

ΔA_S - changes in the absorption at the singlet maximum

Quantum yield of intersystem crossing (Φ_{ISC}) was calculated using comparative method according to the equation:

$$\Phi_T^P = \Phi_T^R \frac{\Delta A_T^P}{\Delta A_T^R} \frac{\varepsilon_T^R}{\varepsilon_T^P} \quad \text{(S3)}$$

Φ_T^P- ISC quantum yield of the tested porphyrins

Φ_T^R- ISC quantum yield of the reference compound

ΔA_T^P- changes in the triplet absorption of the tested porphyrin

ΔA_T^R- changes in the triplet absorption of the reference compound

ε_T^P- triplet excited state molar absorption coefficient of the tested porphyrin

ε_T^R- triplet excited state molar absorption coefficient of the reference compound

Quenching rate constant of a triplet excited state (k_q) by molecular oxygen was calculated using the Stern-Volmer equation:

$$\frac{1}{\tau} = \frac{1}{\tau_0} + k_q\,[O_2] \qquad \text{(S4)}$$

τ- lifetime of a triplet excited state in the presence of a quencher

τ_0- lifetime of a triplet excited state without the presence of a quencher

k_q – quenching rate constant ($M^{-1}s^{-1}$)

Area under the curve (AUC) of the photodegradation of DPBF in presence of porphyrins was calculated according the formula:

$$AUC = \frac{\left(\left(I/I_0\right)_s + \left(I/I_0\right)_f\right)}{2\,(t_f - t_s)} \qquad \text{(S5)}$$

$(I/I_0)_s$ – ratio of the fluorescence intensity and initial fluorescence intensity at the beginning of the measurement interval

$(I/I_0)_f$ - fluorescence intensity and initial fluorescence intensity ratio at the end of the measurement interval

t_s – time at the beginning of the interval of the measurement

t_f - time at the end of the interval of the measurement

2.2.TC-SPC and LFP spectra for porphyrinsTOPyP3-CH$_3$ and TOPyP3-C$_{17}$H$_{35}$

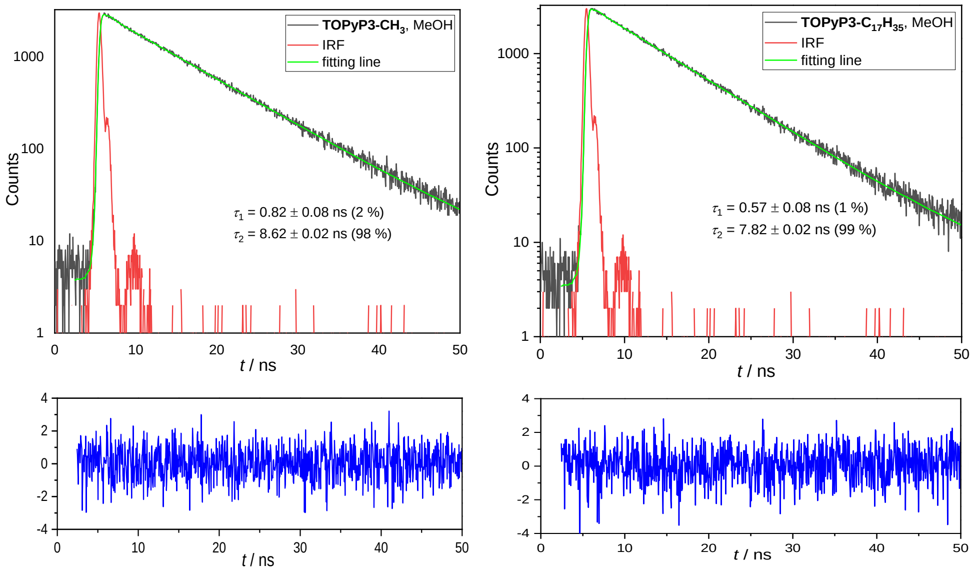

Figure S8. Fluorescence decay of porphyrin **TOPyP3-CH$_3$** (left) and **TOPyP3-C$_{17}$H$_{35}$** (right) in MeOH (N_2) obtained using TC-SPC (black line) and the fit to a sum of two exponents (green

line). λ_{ex} = 405 nm, A_{405nm}(TOPyP3-CH$_3$) = 0.096; A_{405nm}(TOPyP3-C$_{17}$H$_{35}$) = 0.11. The bottom panel of the figures correspond to the weighted residuals between the experimental and the fitted values.

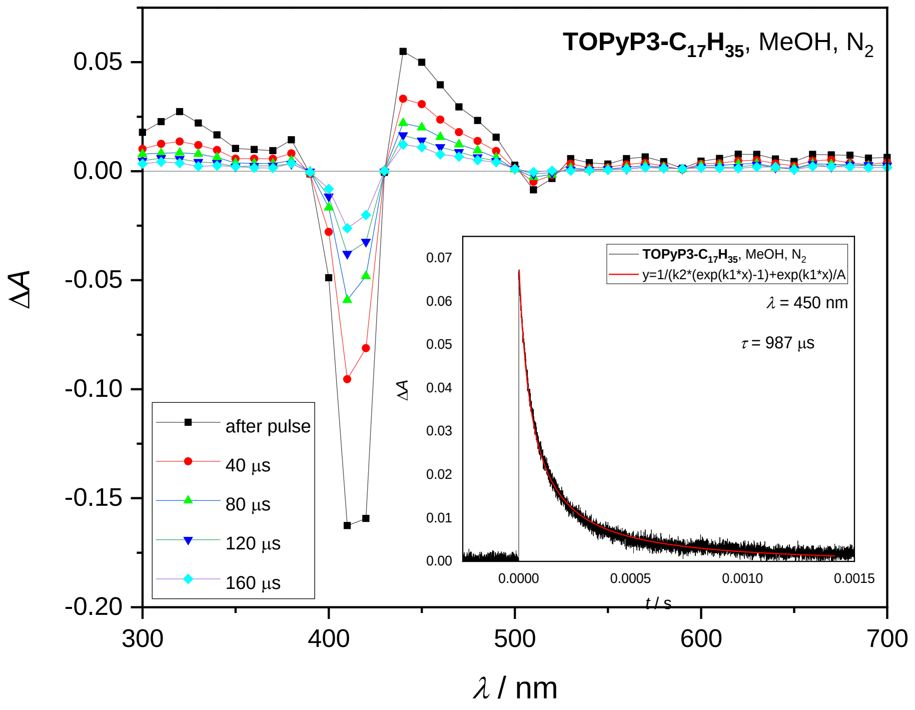

Figure S9. Transient absorption spectra after 355 nm laser excitation of **TOPyP3-C$_{17}$H$_{35}$** in MeOH purged with N$_2$. $A_{355\ nm}$ = 0.21, $E_{355\ nm}$ = 3 mJ. Inset: transient absorption kinetics at 450 nm after 355 nm laser excitation of porphyrin **TOPyP3-C$_{17}$H$_{35}$**.

3. Western blot analysis for HIF-1α stabilisation

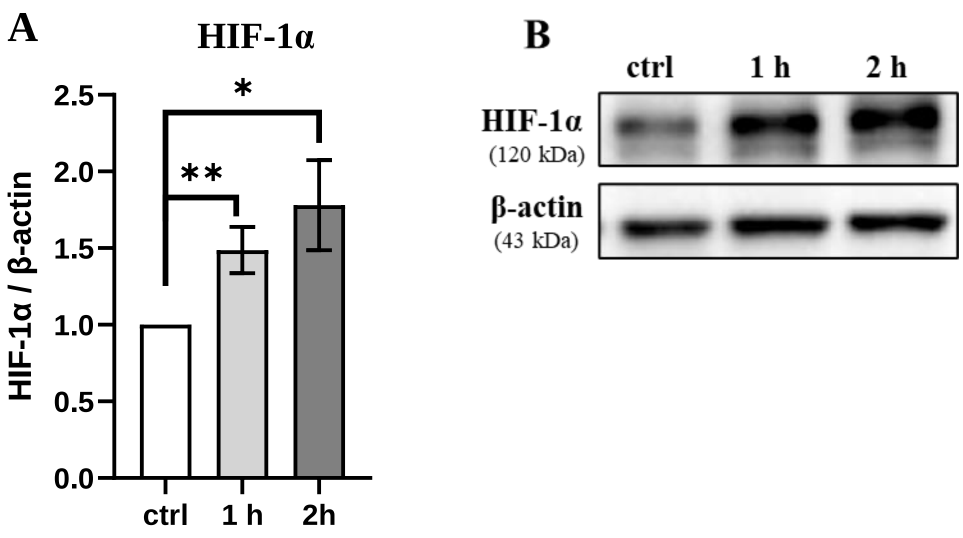

Figure S10. HIF-1α protein levels after incubation with 100 μM CoCl$_2$, hypoxia mimetic agent. Bar charts show the densitometric analysis of HIF-1α protein levels normalized to β-actin in A375 cell line (A) and one western blot image as an example of the results (B). Results were

shown as an average of measurement in triplicate with SEM in error bars. Control represents the cells without addition of $CoCl_2$. Statistical analysis was done using standard *t*-test in comparison to the control. Significance was $p < 0.0001$ and it was shown using the following signs: **** < 0.0001; 0.0001 <*** < 0.001; 0.001 < ** < 0.01; 0.01 < * < 0.1; ns > 0.1 (not significant).

4. Cellular uptake of (oxidopyridyl)porphyrins (calibration curves and temperature-dependent cellular uptake

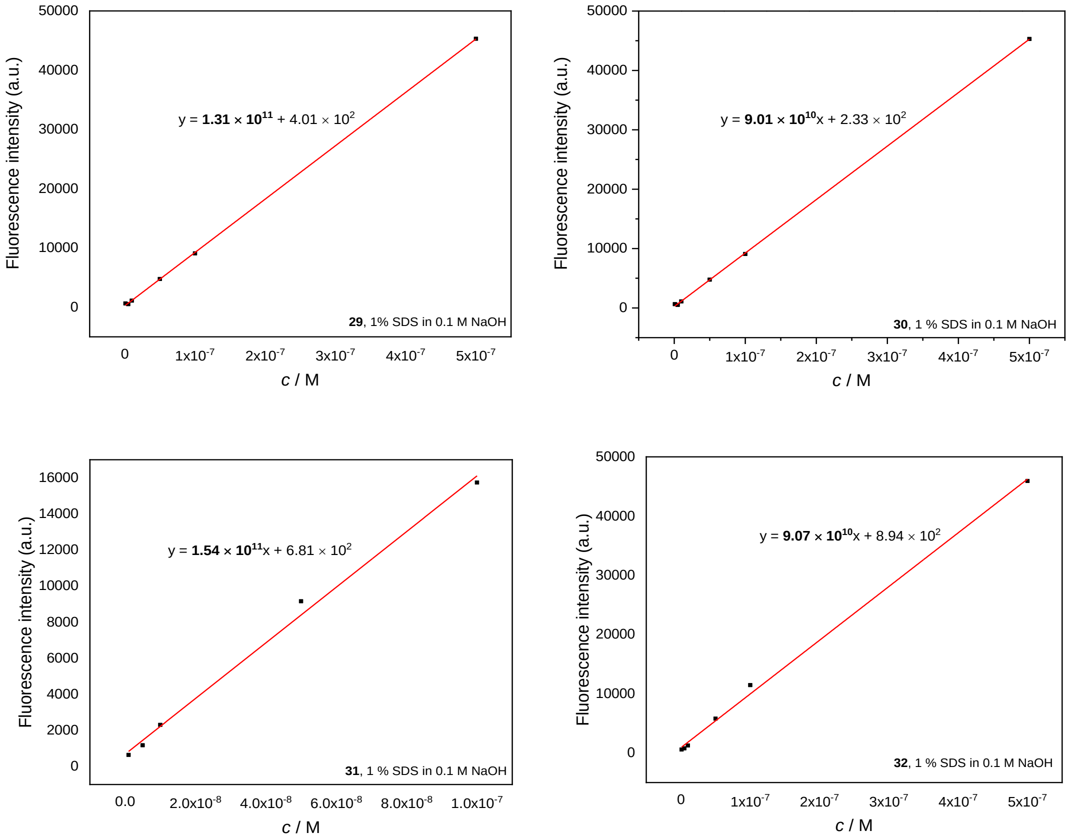

Figure S11. Calibration curves in 1% SDS in 0.1 M NaOH used for calculation of concentration of (oxidopyridyl)porphyrins substituted with an alkyl chain of different length (up: **TOPyP3-CH** (left), **TOPyP3-C_9H_{19}** (right); down: **TOPyP3-$C_{13}H_{27}$** (left), **TOPyP3-$C_{17}H_{35}$** (right)) in the experiments of cellular uptake.

A

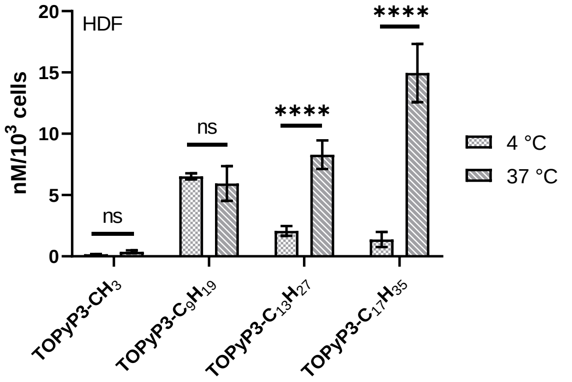

37 °C	$-CH_3$	$-C_9H_{19}$	$-C_{13}H_{27}$	$-C_{17}H_{35}$
$-CH_3$				
$-C_9H_{19}$	****			
$-C_{13}H_{27}$	****	**		
$-C_{17}H_{35}$	****	****	****	

B

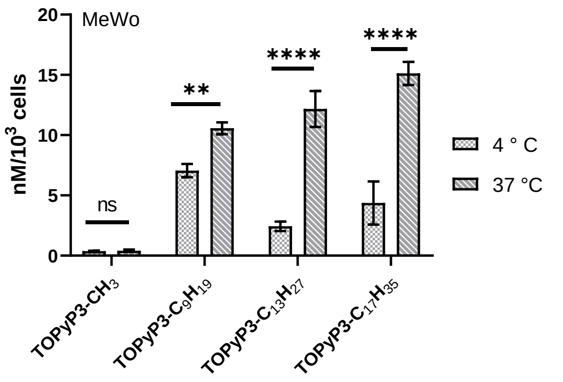

37 °C	$-CH_3$	$-C_9H_{19}$	$-C_{13}H_{27}$	$-C_{17}H_{35}$
$-CH_3$				
$-C_9H_{19}$	****			
$-C_{13}H_{27}$	****	**		
$-C_{17}H_{35}$	****	****	****	

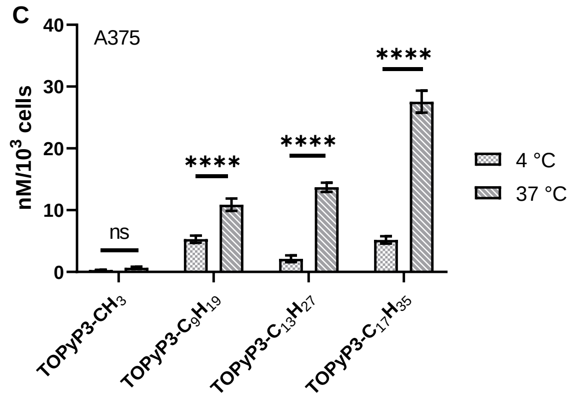

37 °C	**-CH$_3$**	**-C$_9$H$_{19}$**	**-C$_{13}$H$_{27}$**	**-C$_{17}$H$_{35}$**
**-CH$_3$**				
**-C$_9$H$_{19}$**	****			
**-C$_{13}$H$_{27}$**	****	ns		
**-C$_{17}$H$_{35}$**	****	****	****	

Figure S12. Comparison of the cellular uptake at 4 °C and 37 °C after incubation for 24 hours with (oxidopyridyl)porphyrins in HDF (A), MeWo (B) and A375 (C) cell line. All results are shown as a mean concentration per 10^3 cells (nM) with standard deviations as error bars. Statistical analysis of cellular uptake at different temperatures (statistics presented on the graph) and the differences on the cellular uptake at 37 °C based on the alkyl chain length (table statistics) was done using two-way ANOVA with Tukey *post-hoc* test. Significance was $p < 0.0001$ and it was shown using the following signs: **** < 0.0001; 0.0001 <*** < 0.001; 0.001 < ** < 0.01; 0.01 < * < 0.1; ns > 0.1 (not significant).

5. (Photo)cytotoxicity of (oxidopyridyl)porphyrins on different cell lines

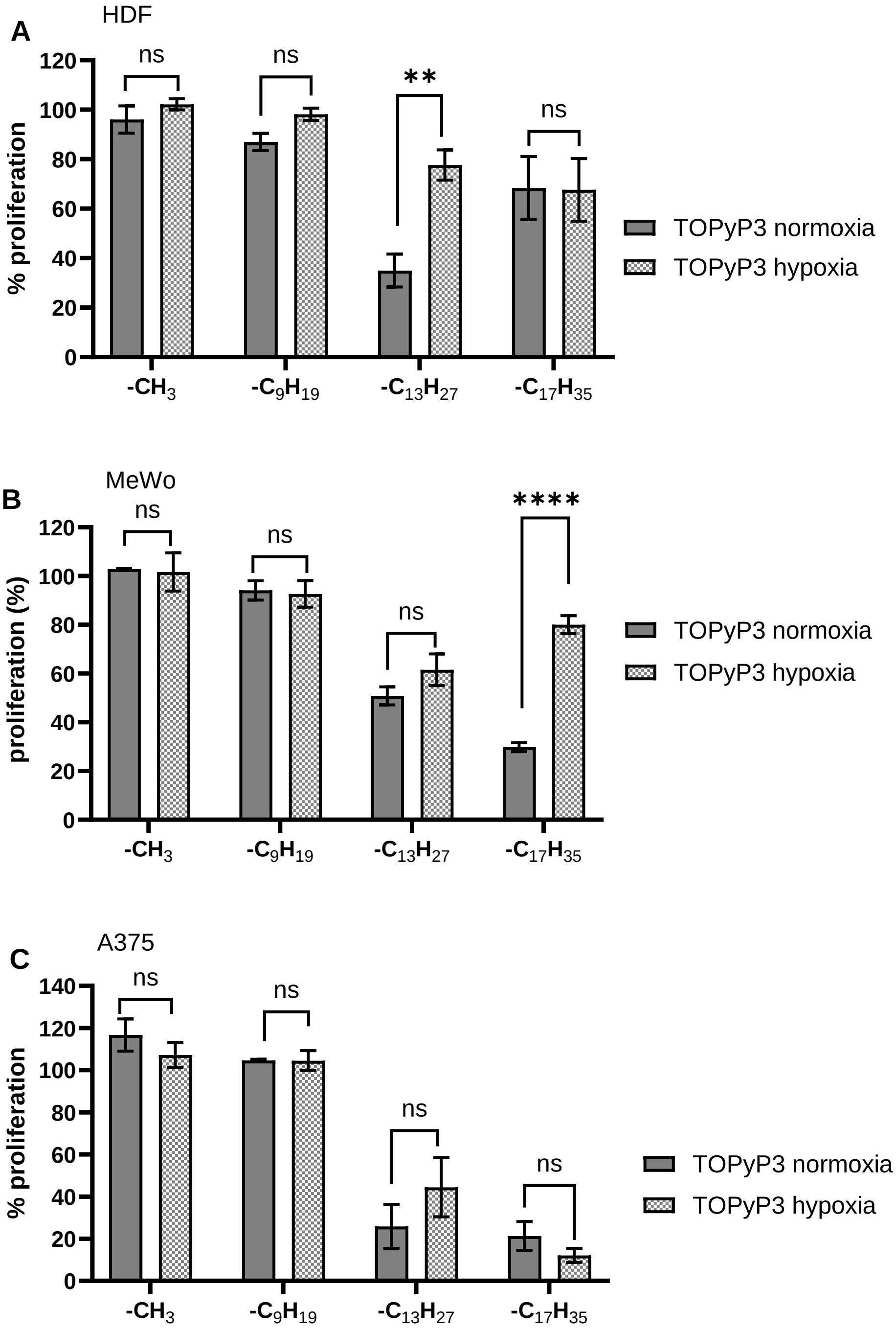

Figure S13. Comparison of the proliferation at 1 µM concentration of the (oxidopyridyl)porphyrin (**TOPyP3**) with an alkyl chain of 1, 9, 13 and 17 C atoms in the conditions of normoxia and $CoCl_2$-induced hypoxia on HDF (A), MeWo (B) and A375 (C) cell line. Results are presented as an average of three individual measurements and error bars are representing standard error. Statistical analysis was performed using two-way ANOVA with Tuckey *post-hoc* test. Significance was $p < 0.0001$ and it was shown using the following signs: **** < 0.0001; 0.0001 <*** < 0.001; 0.001 < ** < 0.01; 0.01 < * < 0.1; ns > 0.1 (not significant).

Table S1. Calculated selectivity index (SI) of melanoma cell lines, determined by dividing the obtained IC_{50} values for fibroblasts (HDF) with IC_{50} values obtained for melanoma cells. All cells were treated with *N*-methylated porphyrins (**TMPyP3**) or (oxidopyridyl)porphyrins (**TOPyP3**) with an alkyl chain of different length (9, 13 and 17 C atoms) and after irradiation for 30 minutes with red light (λ = 643 nm, 2 mW/cm^2, total light dose 3.6 J/cm^2), under normoxia or $CoCl_2$-induced hypoxia. Stock solutions of porphyrins (20 mM) were prepared in DMSO and diluted in DMEM.

**Porphyrins**		**SI** (IC_{50} (HDF) / IC_{50} (melanoma cells))			
		**MeWo**		**A375**	
		No $CoCl_2$	100 µM $CoCl_2$	No $CoCl_2$	100 µM $CoCl_2$
**TMPyP3**	**-C_9H_{19}**	2.0	1.3	10.6	9.0
	**-$C_{13}H_{27}$**	1.9	1.6	5.4	4.5
	**-$C_{17}H_{35}$**	1.0	1.0	2.2	2.0
**TOPyP3**	**-C_9H_{19}**	0.6	1.2	0.9	1.4
	**-$C_{13}H_{27}$**	0.8	1.5	1.1	3.0
	**-$C_{17}H_{35}$**	2.0	1.6	1.7	1.2
